# Impact of various heterocyclic π-linkers and their substitution position on the opto-electronic attributes of the A–π–D–π–A type IECIO-4F molecule: a comparative analysis†

**DOI:** 10.1039/d2ra04097b

**Published:** 2022-07-20

**Authors:** Sahar Javaid Akram, N. M. A. Hadia, Javed Iqbal, Rana Farhat Mehmood, Saleem Iqbal, Ahmed M. Shawky, Areeba Asif, H. H. Somaily, Muhammad Raheel, Rasheed Ahmad Khera

**Affiliations:** Department of Chemistry, University of Agriculture 38000 Faisalabad Pakistan javedkhattak79@gmail.com javed.iqbal@uaf.edu.pk rasheedahmadkhera@yahoo.com rasheed.ahmad.khera@uaf.edu.pk; Physics Department, College of Science, Jouf University P.O. Box 2014 Sakaka Al-Jouf Saudi Arabia nmhadia@ju.edu.sa; Department of Chemistry, Division of Science and Technology, University of Education Township Lahore 54770 Pakistan; Department of Chemical Engineering, Wah Engineering College, University of Wah Quaid Avenue 47040 Wah Cantt Pakistan; Science and Technology Unit (STU), Umm Al-Qura University Makkah 21955 Saudi Arabia; Research Center for Advanced Materials Science (RCAMS), King Khalid University Abha 61413 P.O. Box 9004 Saudi Arabia; Department of Physics, Faculty of Science, King Khalid University P.O. Box 9004 Abha Saudi Arabia; Department of Chemistry, Baluchistan University of Information Technology, Engineering and Management Sciences (BUITEMS) Quetta 87300 Pakistan

## Abstract

To investigate the consequence of different substitution positions of various π-linkers on the photovoltaic properties of an organic solar cell molecule, we have introduced two series of six three-donor molecules, by the substitution of some effective π-linkers on the A–π–D–π–A type reference molecule IECIO-4F (taken as IOR). In series “a” the thienyl or furyl bridge is directly linked between the donor and acceptor moieties, while in series “b” the phenyl ring of the same bridge is working as the direct point of attachment. The frontier molecular orbitals, density of states, transition density matrix, molecular electrostatic potential surfaces, exciton binding energy, excitation energy, wavelength of maximum absorption, open-circuit voltage, fill factor, and some other photovoltaic attributes of the proposed molecules were analyzed through density functional theory (DFT) and its time-dependent (TD) approach; the TD-DFT method. Though both series of newly derived molecules were a step up from the reference molecule in almost all of the studied characteristics, the “a” series (IO1_a_ to IO3_a_) seemed to be better due to their desirable properties such as the highest maximum absorption wavelength (*λ*_max_), open-circuit voltage, and fill factor, along with the lowest excitation and exciton dissociation energy, *etc.* of its molecules. Also, the studied morphology, optical characteristics, and electronic attributes of this series of proposed molecules signified the fact that the molecules with thienyl or furyl ring working as the direct link between the acceptor and donor molecules showed enhanced charge transfer abilities, and could provide a maximum quantum yield of the solar energy supplied.

## Abstract 2

We have introduced two series of six three-donor molecules, by the substitution of some effective π-linkers on the A–π–D–π–A type reference molecule IECIO-4F (taken as IOR) for efficient organic solar cells.

## Graphical abstract

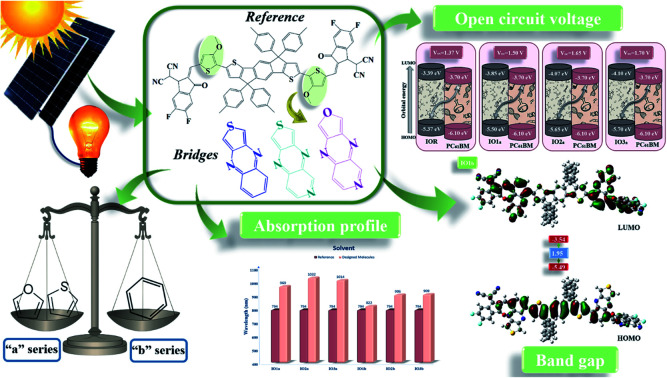

## Introduction

Due to the increasing global energy crisis, the demand for renewable energy supplies has increased tremendously. Amongst the different sustainable energy sources, solar energy seems to be the most convenient and efficient.^1^ For decades, crystalline wafer-based silicon solar cells have been utilized to generate electricity from solar energy. These photovoltaic cells are expensive and brittle, and in addition, they have a low absorption range.^2^ In contrast, their counterparts, thin-film organic solar cells (OSCs), have proved themselves to be superior due to their facile and low-cost fabrication attained through different solution processing techniques. Moreover, they are light weight, mechanically flexible, semi-transparent, and have easily tunable energy gaps.^3,4^ However, their photo-conversion efficiency (PCE) is 18–20% lower than inorganic (silicon) solar cells. Thus numerous efforts have been made by scientists all around the globe in order to overcome this drawback.^5,6^

The most recent of the many types of organic photovoltaic cells are the small molecule-based bulk-heterojunction (BHJ) OSCs. The molecules utilized in these solar cells have a definite weight and structure, high purity, and are also easily reproducible. The active layer of these significant OSCs is a blend of various donor and acceptor molecules, where donor molecules act as p-type, while acceptors act as n-type semiconductors.^7,8^ A necessary condition for efficient intramolecular charge transfer is proper differentiation of acceptor and donor regions. This way, the charge, after its generation in the donor region, can easily transfer within the molecule toward the acceptor region.^9^ Sometimes, the presence of a bridge between these donor and acceptor regions can significantly improve the intramolecular charge transfer. These bridges increase the conjugation in the molecule leading to a high fill factor and an increased photocurrent, owing to the reduced charge recombination in the molecule.^10^ In contrast to the molecules with no spacer, the molecules with a prominent spacer present, have significantly improved planarity and π–π interactions. In addition, upon stacking, they seem to suppress the aggression between them, owing to their increased conjugation length.^11^ Though various studies have been performed on the effect of different π-linkers on the photovoltaic performance of the OSCs, the data still seems to be lacking as compared to that present out there on the effect of different acceptor or donor regions.^12^

To increase the charge separation and ease of intramolecular charge transfer, various thiophene, furan, oligothiophene, polyenes, and other fused aromatic rings have been introduced into the otherwise donor–acceptor (D–A) type molecule. Photovoltaic molecules with thiophene derivatives as π-linkers have demonstrated efficient device performance in OSCs.^13^ For example, alkoxy-thiophene bridges were incorporated as π-spacers into IEIC molecule by Li Jianfeng *et al.*, and a significant improvement in the optoelectronic properties of the resulting IEICO molecule was seen.^14^ In our research work, IEICO-4F was chosen as the reference molecule due to its promising optoelectronic and photovoltaic attributes cited in many literature. This molecule consists of an electron-rich indacenodithiophene (IDT) based donor core, which has four phenyl rings contributing to its electron-rich character. Attached to this donor core on both sides are the methoxy thiophene π-linkers that act as prominent charge transfer moieties for efficient charge transfer from the donor core to the peripheral acceptors. The acceptors attached at the peripheries of this molecule are the strongly electron withdrawing 2-(5,6-difluoro-2-methylene-3-oxo-indan-1-yl)-malononitrile groups. Accredited to these proficient fragments of this highly planar molecule, it shows significant absorption in the chloroform solvent with a range of 600–900 nm, locating close to the near-infrared region, and a narrow bandgap of only 1.73 eV.^15^ In addition, a blend of this molecule with PBDTTT-EFT donor molecule exhibited an impressive experimental power conversion efficiency of 10%, an open-circuit of voltage of 0.739 V with a notable short-circuit current of 22.8 mA cm^−2^ and an energy loss of only 0.501 eV.^15^ Furthermore, IEICO-4F, by promoting the crystallization of the film, reduces the charge recombination of the corresponding device.^16^ So, taking into account these remarkable attributes of IEICO-4F, this molecule was selected for our research in a quest to increase its already outstanding optoelectronic properties for prominent organic photovoltaic cells.

**Fig. 1:**
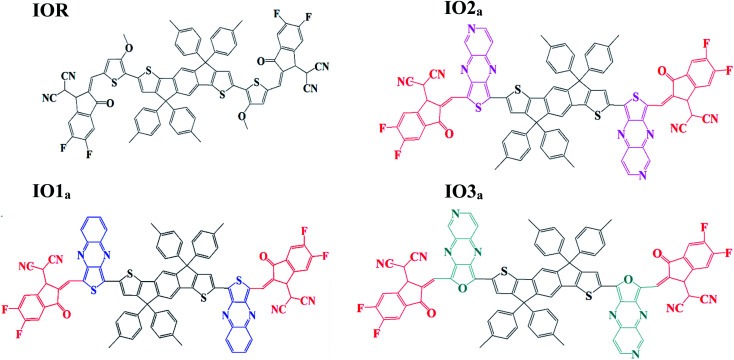
Pictorial representation of reference (IOR) and “a series” of all the scrutinized molecules (donor core is colored black, acceptors are red in colour, while blue, pink, and green represents various bridges).

Liu *et al.* studied the effect of the substitution position of arylamine moieties on thiophene π-linkers. It was seen that the 2,5-position demonstrated a 40% increase in PCE as compared to the 3,4-position.^17^ It is generally seen that the point of attachment of the bridges between the donor and acceptor region of a molecule remarkably affects the hole mobility, electrochemical attributes, and photovoltaic performance of the molecule.^18^ Thus, two series (“a” and “b”) of donor molecules are designed here, to study the effect of the substitution position of various π-linkers on the device performance of the molecule under consideration. The spacers in the cited molecule IEICO-4F^19^ were replaced with three new bridges at different sites of attachment (either six-membered or five-membered rings). The bridges utilized for our research were thieno[3,4-*b*]quinoxaline (IO1), 2-thia-4,6,9-triaza-cyclopenta[*b*]naphthalene (IO2), and 2-oxa-4,6,9-triaza-cyclopenta[*b*]naphthalene (IO3). Fig. S1† illustrate the ChemDraw sketches of “b” series of designed molecules, while the rest of the molecules (reference and “a” series) are depicted in Fig. 1. Thus, six new molecules are reported, with two molecules (a and b) having only the difference in point of attachments. The bridges employed for the design of the newly proposed molecules were seen to give prominent results in the literature. These bridges have a slight difference with respect to the substitution of an atom or two, for example in IO2, the carbon of the second phenyl ring is replaced with a nitrogen, similarly, in IO3, the sulphur of the five membered ring is substituted with an oxygen atom.^20^

## Computational details

All the investigated molecular structures were first sketched through ChemDraw 7.0.^21^ Then with the help of these sketches, the molecules were designed in GuassView 6.0 (ref. 22) program, the very same program through which the results obtained after quantum chemical simulations from Gaussian 09 (ref. 23) programs were visualized. After careful examination of the value of maximum absorption (*λ*_max_) of reference molecule IOR, the MPW1PW91 functional, in combination with the 6-31G(d,p) basis set, was selected for all the proceedings in this research work. Also, the spin was restricted for all the computations to avoid any spin contamination in the proposed molecules.

Here, all the six newly designed structures were optimized at their ground state through the above-stated density functional theory (DFT)^24^ level of theory. Then, their molecular dynamics, such as frontier molecular orbitals (FMOs) and molecular electrostatic potential (MESP) surfaces, were assessed from the thus obtained geometrically optimized structures. The density of states (DOS) of these structures was also evaluated to authenticate the results of FMOs. These states were plotted in the form of graphs by the utilization of the PyMOlyze 1.1 (ref. 25) program. Moreover, the excited state properties (maximum absorption wavelength, excitation energy, oscillator strength, *etc.*) of these structures in the gas and solvent (chloroform) phase were also examined through time-dependent DFT (TD-DFT).^26^ The polarizable continuum model (PCM)^27^ model was employed to simulate the solvent environment, and the reason behind the selection of chloroform solvent was its utilization in the cited literature for the reference molecule.^28^ Moreover, the results of maximum absorption were envisioned with the help of graphs obtained from Origin 6.0 software.^29^

Reorganization energy (*λ*_h_ for the hole and *λ*_e_ for the electron) is an important parameter to determine the mobility of electrons or holes in the molecule after their separation. Thus, this characteristic of the molecules was also calculated according to eqn (1) and (2) from Marcus' theory, given below^30,31^

1 *λ*_e_ = [*E*^−^_0_ − *E*_−_] + [*E*^0^_−_ − *E*_0_]

2 *λ*_h_ = [*E*^+^_0_ − *E*_+_] + [*E*^0^_+_ − *E*_0_]

Here, *E*_0_, *E*_+_, and *E*_−_ are the single point energies of the neutral molecule, cation, and anion, sequentially. *E*^−^_0_ is the ground state energy of anion, and *E*^+^_0_ is the ground state energy of cation. While, *E*^0^_−_ and *E*^0^_+_ are the neutral energies of anion and cation, respectively.

The transition density matrices of the cited and newly designed molecules were procured through Multiwfn 3.6 application software.^32^ Some chemical descriptors like band gap, softness, and hardness of the newly derived structures at their ground state were also computed. Furthermore, the binding energy was assessed in order to estimate the minimum amount of energy required to separate the geminate (electron–hole pair) produced in the scrutinized structures after the absorption of radiations. Finally, the open-circuit voltage and fill factor of the studied molecules were calculated to give a direction about the photo-conversion efficiency (PCE) of these molecules, which is a significant parameter to determine the plausibility of the studied molecules in organic solar cells.

## Results and discussion

Generally, alkyl chains have little to no effect on the spectral and optoelectronic properties of a molecule. Thus, for the sake of convention, the alkyl chains of the cited molecule IEICO-4F were substituted with methyl chains, and the molecule thus formed was named IOR. This reference molecule IOR was first examined theoretically through the density functional theory (DFT) method and its time-dependent approach in order to speculate the functional to be utilized for all the executed simulations in this research. For this purpose, firstly, the geometry optimization of the reference molecule IOR was performed at four extensively used and reliable functionals. The functionals utilized here were B3LYP,^33^ CAM-B3LYP,^34^ ωB97XD,^35^ and MPW1PW91.^36^ From there on, the values of bandgaps from all these functionals were compared to that of the experimental bandgap (1.73 eV) cited in the literature.^28,37^ The values of bandgaps attained from the afore-stated functionals are tabulated alongside the experimental one in Table 1.

**Table 1:** Comparing the computed bandgaps from four analyzed functionals to the cited bandgap

Functionals	Computed bandgap	Cited bandgap
B3LYP	1.46	1.73
CAMB3LYP	2.05	—
ωB97XD	2.16	—
MPW1PW91	1.56	—

**Fig. 2:**
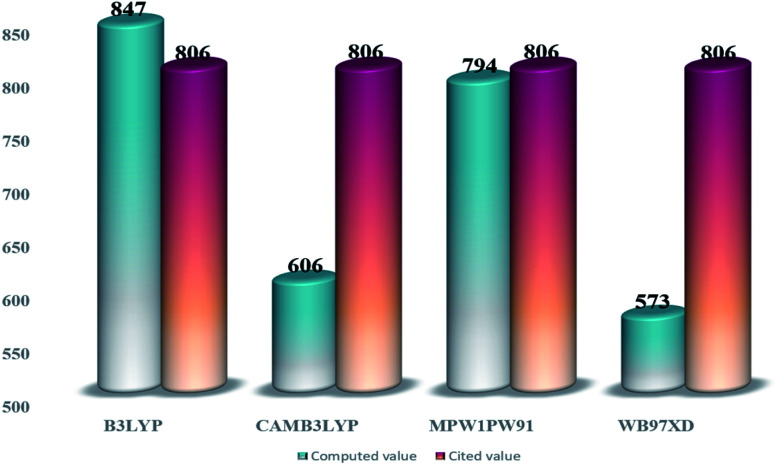
Comparative bar graph illustrating *λ*_max_ from different functionals and their closeness to the cited value of IOR.

From the tabulated data, the bandgap from MPW1PW91 seems to be the closest one to the experimental value. Thus for ground state optimization of geometries, this functional was selected as the one. Furthermore, for the selection of the functional for the excited state properties, all the afore-stated functionals were evaluated for their wavelengths of maximum (*λ*_max_) absorption in the cited chloroform solvent. The values of maximum absorption (*λ*_max_) obtained from all these functionals, at split valence 6-31G(d,p) basis set, were compared with the experimental value (806 nm) of the IOR molecule cited in the literature.^19^ From the values of 847 nm, 606 nm, 573 nm, and 794 nm obtained for the afore-stated functionals, respectively, it is seen that the value from MPW1PW91 is the closest to the reference value (Fig. 2). Thus, from this point onwards, based on both these validations, MPW1PW91 functional was selected, for further computations in this investigation of the structural, photo-physical, and optoelectronic properties of the newly proposed series of donor molecules.

### Structural design consideration

To examine the excited state properties of a molecule, it is crucial to first optimize the molecule at its ground state, so that a proper evaluation or comparison between the ground and excited state properties can be performed. Thus, all the investigated structures were first optimized at their ground state, and then their bond parameters (*i.e.*, bond length and dihedral angle) were analyzed in order to study the variation in them caused due to the various bridges and their substitution positions.

**Fig. 3:**
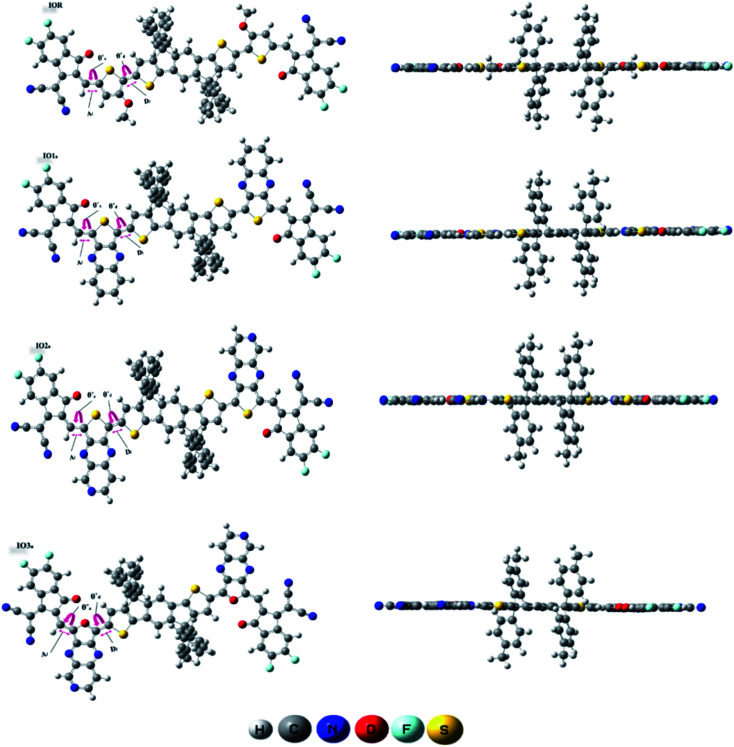
GaussView structures of IOR and “a” series of the all the newly reported molecules.

The bond parameters studied for the optimized molecular structures are enlisted in Table S1,† and they are also pictorially illustrated in Fig. 3 (for reference and “a” series of designed molecules) and Fig. S2† (for “b” series of newly formulated molecules). The bond length between the substituted π-linker and the attached acceptor moieties is demonstrated as *A*_l_, while the one between donor and π-linker is written as *D*_l_. It was observed that the bond lengths between carbon atoms for all the studied point of attachments were within the range of the single bond length of 1.54 Å and double bond length of 1.34 Å. And it is commonly known that the closer a bond length is to the carbon–carbon double bond length, the more conjugated the corresponding molecule will be. This conjugation would help in better charge transfer and could significantly improve the opto-electronic properties of the evaluated molecules.^38^ Between our “a” and “b” series, all the three molecules of the “a” series established a decrease in their *A*_l_ and *D*_l_ bond length, while a prominent increase in the studied bond lengths of molecules of the “b” series was seen. The decreased bond length in molecules of “a” series could contribute to their enhanced charge transfer attributes and absorption ranges due to their enhanced conjugation than that of their counterparts. Furthermore, in comparison to the reference molecule, the molecules of the “a” series seem to have lower values of evaluated bond lengths. While that of the “b” series have higher values and this illustrates the more enhanced conjugation in molecules of the “a” series than the reference IOR molecule and also hints toward the somewhat lowered conjugation in the molecules of the “b” series. The decreased conjugation in the studied bond length of molecules of the “b” series could be their twisted confirmation, as evaluated from their dihedral angles below.

The dihedral angle for the specified attachment sites in Fig. 3, (for reference and “a” series of designed molecules) and Fig. S2† (for “b” series of newly formulated molecules), was also examined in order to study the planarity in the molecules. The lower the dihedral angle a molecule has, the more planarity it will have. A general perspective is that planar molecules having bulky electron donor and acceptor segments are crucial in attaining effective separation and generation of charges. This planar geometry could also increase the conjugation length, as well as enhance the long-range π–π stacking in the molecule.^39^ In the studied molecules, the dihedral angle on the acceptor side, is represented through *θ*_a_, and the one on the donor side, is written as *θ*_d_. The *θ*_a_ and *θ*_d_ of all the molecules of the “a” series were lower than their counterparts in the “b” series. Moreover, these dihedral angles of all the molecules of the “a” series, except for *θ*_a_ of IO3_a_, were comparable to or lower than that of the reference molecule IOR, which shows the greater planarity in these molecules as compared to the reference molecules. This planarity could only be attributed to the fact that in the “a” series, all the acceptors and donors are attached through a thiophene/furan ring and not a bulky phenyl ring, as is the case in the “b” series. So, it could be supposed that molecules of “a” series, just like the reference molecule, due to the high level of planarity in their structures, could show better charge transfer attributes than that of the molecules of the “b” series.

### Electronic properties

The highest occupied molecular orbitals (HOMO) and the lowest unoccupied molecular orbitals collectively constitute the frontier molecular orbitals (FMOs), and the difference between them is commonly known as the electronic band gap (*E*_g_).^40^ Generally, for a high performing photovoltaic chromophore, the HOMO should be concentrated over the donor region of a molecule, while the LUMO should be more concentrated over the acceptor region. This way, the electron from the donor region could effectively be transferred over to the acceptor region.^41^ Also, the presence of charge density on the bridges show their contribution to the charge transfer from HOMO towards LUMO. Moreover, the lesser the band gap between the FMOs, the more efficient will be the active layer made from these molecules.^42^

The orbital density analysis from Fig. S3† reveals that in the reference molecule IOR, the HOMO is spread over the donor, bridge, and a little bit on the acceptor region of the molecule. On the other hand, its LUMO is spread over the whole molecule except for the perpendicular phenyl rings in the central core. A similar trend of spread of charge density is followed by all the three molecules of the “a” series. This trend could be due to the similar planar topology of the reference and “a” series molecules. In the case of the “b” series, the HOMO charge density is highly concentrated over the central donor and π-linkers, and the LUMO is more densely spread over the π-linkers along with the acceptors. The lower charge density over the acceptors in the case of HOMO and donor in the case of LUMO could be due to the distorted configuration of the molecules in the “b” series.

So, here we could say that the molecules in the “b” series could act as better-performing photovoltaic molecules than the “a” series. But the spread of charge density over the whole molecule in “a” also signifies the presence of effective conjugation in them, which is an significant parameter in effective photovoltaic chromophores. The perpendicular orientation of the phenyl rings in the central core of the molecule could be the reason behind the no charge spread over them in both the HOMO and the LUMO. While comparing with the reference molecule, the trend of spread of charge density in bridges for FMOs of reference molecule was seen to be a bit different from all the newly proposed ones, as the HOMO charge density for all the newly reported molecules was minutely distributed over their bulky π-linkers, but this was not the case in IOR, where the charge density was actually significantly present on the alkoxy thiophene bridges.

The values of the evaluated FMOs and their calculated electronic band gap are tabulated in Table 2. It can be seen that the electronic band gap (*E*_g_) for all the newly derived molecules is lower than the reference molecule. Also, from the values of FMOs, it was seen that the HOMO is lower-lying in the newly derived molecules than in the reference molecule, and this illustrates their better stability than IOR. This signifies the better performing capabilities of all our reported molecules than the IOR in the organic photovoltaic cells. Upon comparison between the *E*_g_ of both the series under consideration, the molecules in the “a” series exhibited a much lower band gap as compared to their counterparts in the “b” series. This lower *E*_g_ in the molecules of the “a” series could enhance the charge transfer between their FMOs.

**Table 2:** Values of evaluated FMOs and the calculated band gap between them

Molecules	*E*_H_ (eV)	*E*_L_ (eV)	*E*_g_ (eV)
IOR	−5.37	−3.39	1.98
IO1_a_	−5.50	−3.85	1.65
IO2_a_	−5.65	−4.07	1.58
IO3_a_	−5.70	−4.10	1.60
IO1_b_	−5.49	−3.54	1.95
IO2_b_	−5.50	−3.72	1.78
IO3_b_	−5.53	−3.74	1.79

Specifically, the lowest bandgap amongst all the analyzed molecules of IO2 (both a and b). The reason behind this narrow bandgap could be due to the presence of its highly effective π-linkers, which though is quite similar to the bridges present in IO3, differing only in the presence of sulphur atom in place of oxygen, and this sulphur atom due to its lower ability to attract the electron towards itself as compared to the oxygen atom could effectively transfer charge from donor to terminal acceptor, instead of retaining it, thus lowering the bandgap. The highest bandgap within both the proposed series was of IO1_a_, which could be ascribed to the absence of one of the electron-withdrawing nitrogen atom at its π-linkers, as compared to the other two bridges. But overall, till now both the molecules in the “a” and the “b” series could act as better candidates for the active layer in the constructive organic solar cells due to their lower bandgaps than IOR.

### Quantum chemical indices

In order to study the charge-transfer properties of any molecules, its softness, as well as hardness, could be analyzed.^43^ The chemical hardness is basically half of the difference between the ionization potential (IP) and electron affinity (EA) values. These values are also quite notable in determining the HOMO and LUMO energy levels of a molecule, as a low-lying HOMO exhibits high values of IP, and a higher value of EA proclaims a high LUMO.^44^ These values for the molecules under study calculated from eqn (3) and (4) are demonstrated in Table S2.†

3 IP = [*E*^+^_0_ − *E*_0_]

4 EA = [*E*_0_ − *E*^−^_0_]

Here, *E*^+^_0_ and *E*^−^_0_ are the ground state energies of cation and anion, respectively, obtained from their optimized geometries. While, *E*_0_ is simply the ground state energy of the neutral molecule. The higher IP values of the newly reported molecules discloses their low-lying HOMO. Similarly, their higher EA values as compared to the IOR exhibits their higher LUMO.

The relation of IP and EA with the chemical hardness and softness could be seen from the eqn (5) and (6).^45^5
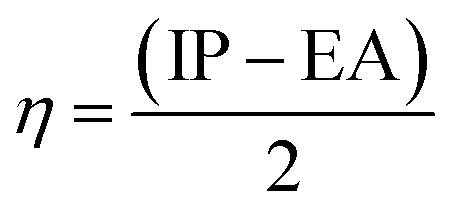
6
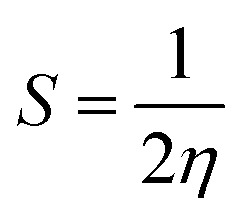
Here, *η* is the chemical hardness. While *S* is the softness of the molecule. This softness is the reciprocal of twice the hardness.^46^ So, both these values are in counter relation with one another. For a highly efficient charge transfer in an OSC, the *η* should be lower and value of *S* should be higher. From the values of both these parameters, enlisted in Table S2,† it can be seen that though all our designed molecules follow this trend, the molecules in the “a” series show a remarkable decrease in the value of hardness and increase in the value of softness, as compared to the IOR. This trend of both the evaluated parameters signifies the enhanced ability of molecules of “a” series to easily transfer charge than both the reference and the “b” series molecule. The reason behind this lowered hardness and higher softness values of molecules of “a” series, could be the relative position of the bulky bridges in the overall structure of the molecule. So concisely, these molecules could exhibit better charge transfer attributes than those of the “b” series ones and the reference molecule, with IO1_a_ having the best attribute to do so. Even in the “b” series, IO1_b_ showed the most favorable character among all, which could be endorsed to the presence of the electron transporting proficient π-bridges in IO1 molecules.

### Density of states

**Fig. 4:**
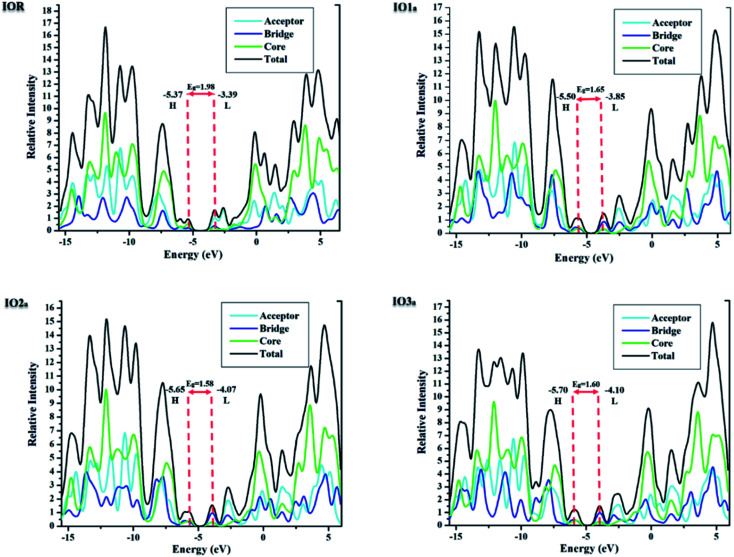
DOS graphs of reference and “a” series of the investigated molecules.

The density of states (DOS) for all the scrutinized molecules was evaluated, in order to validate the results of the FMO analysis. These DOS are actually the number of states that an electron is allowed to occupy at a specific energy level, and they also represent the contribution of HOMO and LUMO orbitals in any electronic excitation.^47^ For the sake of ease of evaluation of the DOS, the molecules were fragmented into donor, acceptor, and bridges. In Fig. 4 and S4,† the partial density of states (PDOS) of acceptors is illustrated through the cyan line, bridges through blue, and that of the core is through the green line. These PDOS collectively formulate the black line, which is called the total DOS (TDOS). Moreover, the peaks to the left of the central planar zone represent the HOMO energy level, and those present towards its right illustrate the LUMO energy levels. While this planar “no peak zone” is actually the band gap between the analyzed energy levels and is in close collaboration with the findings of the value *E*_g_ from FMO analysis. A peak present more towards the LUMO region of the DOS plots illustrates the prominent conductive abilities of the corresponding fragment. In the plots of DOS, as compared to IOR, all the proposed molecules seem to have a high contribution of bridges in their LUMO region, with “a” series having the highest peaks of bridges in their DOS plots. This implies the higher involvement of the bridges in the “a” series to conduct charges.

Table 3, obtained from Mulliken's calculations, helps in the quantitative assessment of the contribution of each fragment to a specific energy level. It is demonstrated that in IOR the contribution of the donor in elevating the HOMO value is higher than the molecules of the “a” series, while its contribution is lower than the molecules of the “b” series. Actually, the spread of charge density in the HOMO, as well as LUMO of the molecules in the “a” series, illustrates their somewhat better planar configuration than the reference molecule. An important point to note here is that the substituted bridges in both the “a” and “b” series have higher charge density than that of the cited molecule in both the studied FMOs, this signifies the contribution of the newly introduced bridges to transfer charges than that of the alkoxy thiophene bridges present in IOR. Furthermore, from the quantitative values of the percentage involvement of all the fragments of the molecules in DOS plots, it can be understood that the newly substituted bridges in the molecules have higher values of their involvement in the LUMO charge density than HOMO, which signifies their enhanced charge conductive abilities.

**Table 3:** Percentage contribution of different fragments of all the newly presented molecules along with the reference molecule (IOR)

Molecules	HOMO			LUMO		
	Donor (%)	Linker (%)	Acceptors (%)	Donor (%)	Linker (%)	Acceptor (%)
IOR	58.1	22.7	19.2	17.5	24.4	58.1
IO1_a_	40.5	30.5	29.0	21.9	56.2	21.9
IO2_a_	42.6	29.8	27.6	19.4	60.0	20.7
IO3_a_	36.6	30.3	33.1	20.8	59.6	19.6
IO1_b_	65.6	24.6	9.80	11.3	51.2	37.5
IO2_b_	68.6	23.6	7.80	9.30	58.1	32.6
IO3_b_	68.7	23.9	7.40	9.20	56.5	34.3

### Spectral absorption and other photovoltaic attributes

**Fig. 5:**
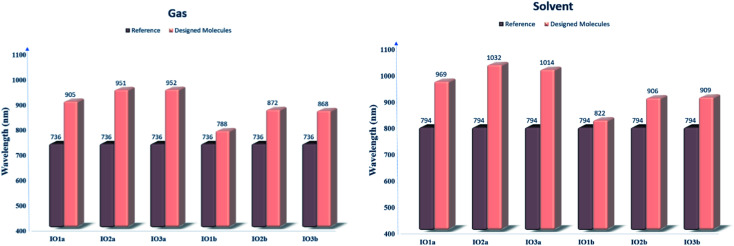
Bar graphs comparing the *λ*_max_ of reference with that of the designed molecules in both the gas (left) and solvent (right) phase.

For the evaluation of the excited state photovoltaic properties of the scrutinized molecules in both the gas and solvent (chloroform) phase, the molecules were computed at the selected functional MPW1PW91 of the TD-DFT calculations. The wavelength of maximum absorption (*λ*_max_) of electromagnetic radiations between the HOMO and LUMO energy levels is pictorially illustrated with the graphs obtained from Origin 6.0 software in Fig. S5.† Moreover, for easy comparison between the *λ*_max_ of the proposed molecules and the reference molecule, their bar graphs are represented in Fig. 5. Also, the values of optical band gap (*E*_x_), oscillator strength (*f*), light-harvesting efficiency, and the percentage contribution of the FMOs in the studied excitation for both the gas and phase are enlisted in Tables 4 and 5, respectively.

**Table 4:** Computed photovoltaic attributes of all the investigated molecules in the gas phase

Molecules	Computed *λ*_max_ (nm)	Experimental *λ*_max_ (nm)	*E*_x_ (eV)	*f*_os_	LHE	Main configuration
IOR	734	797	1.6848	2.8479	0.9986	H to L (70%)
IO1_a_	905		1.3716	2.2417	0.9943	H to L (69%)
IO2_a_	951		1.3043	2.0534	0.9912	H to L (69%)
IO3_a_	952		1.3018	1.6748	0.9789	H to L (69%)
IO1_b_	788		1.5735	1.9338	0.9884	H to L (69%)
IO2_b_	872		1.4218	1.6237	0.9762	H to L (70%)
IO3_b_	868		1.4289	1.6295	0.9765	H to L (70%)

**Table 5:** Computed photovoltaic attributes of all the investigated molecules in the solvent (chloroform) phase

Molecules	Computed *λ*_max_ (nm)	Experimental *λ*_max_ (nm)	*E*_x_ (eV)	*f*_os_	LHE	Main configuration
IOR	794	806	1.5624	3.1403	0.9992	H to L (69%)
IO1_a_	969		1.2798	2.6178	0.9977	H to L (69%)
IO2_a_	1032		1.2010	2.3839	0.9958	H to L (69%)
IO3_a_	1014		1.2229	2.0187	0.9904	H to L (69%)
IO1_b_	822		1.5075	2.2098	0.9938	H to L (69%)
IO2_b_	906		1.3692	1.9186	0.9879	H to L (69%)
IO3_b_	909		1.3635	1.8766	0.9867	H to L (69%)

The absorption spectra in Fig. S5† presents two peaks. Here the higher prominent peaks represent the maximum absorption (*λ*_max_), while the lower smaller peaks represent the presence of π–π stacking in the molecules. The smaller peaks are significantly enhanced in the designed molecules as compared to the IOR molecule, which shows the better π–π stacking in the newly reported molecules than the cited one.^48^ Also, the reference molecule IOR has the lowest *λ*_max_ value amongst all, in both the evaluated phases. This *λ*_max_ follows the ascending order of IOR < IO1_b_ < IO2_b_ < IO3_b_ < IO1_a_ < IO2_a_ < IO3_a_ in the gas phase, and a slightly different trend of IOR < IO1_b_ < IO2_b_ < IO3_b_ < IO1_a_ < IO3_a_ < IO2_a_ is followed in the solvent phase as well. The highest values of *λ*_max_ in molecules of the “a” series signifies the presence of enhanced conjugation in their structures, which could lead to absorption of radiations in the higher wavelength. In addition, the IO2_a_ molecule, due to its prominently charge transferring bulky π-bridges and their relative substitution position, displays the highest *λ*_max_ amongst all in the solvent phase, also the reason behind the marginally higher *λ*_max_ value of IO3_a_ in the gas phase could illustrate the enhanced ability of this molecule to transfer charge than all others in the gaseous medium. Between the gas and the solvent phase, the high values of *λ*_max_ in the latter phase exhibit the compatibility of the investigated molecules with the chloroform solvent.^28^ This way, we could assume that all the reported molecules can be easily used for effective solution manufacturing of OSCs, while IO3_a_ could perform slightly better in the gas phase than in the solvent medium.

The optical band gap, commonly known as the excitation energy (*E*_x_) of a molecule, is the minimum amount of energy required to excite an electron^49^ and is an important asset in determining the capability of the desired molecule to be utilized in the active layer of the OSCs. An effectively performing photovoltaic cell generally needs to have a low value of *E*_x_.^50^ From Tables 4 and 5, it can be seen that the *E*_x_ of all the molecules in both the reported series is lower than the *E*_x_ of IOR, while between the “a” and “b” series, the former exhibits the lower values of *E*_x_. Again, the lower *E*_x_ values of all the proposed molecules than reference could be due to their enhanced charge transferring bridges, as opposed to the ones in IOR, while the lowest value amongst all, of the molecules of “a” series, promotes our claim of this series being a better one due to the substitution position of its prominent bridges. In addition, the lowest *E*_x_ value amongst all the molecules in the evaluated solvent phase was seen for IO2_a_, which could also be the reason behind its highest *λ*_max_ value in this solvent. Actually, all the molecules in both the analyzed phases follow the same trend as the one in the case of their *λ*_max_ values, so it could be said that *λ*_max_ has a direct relation to excitation energy in the case of our studied molecules.^51^

The oscillator strength (*f*) is a prominent dimensionless parameter, which has a direct relation with the light-harvesting efficiency (LHE) of a molecule. This LHE is the ability of a molecule to harvest the energy that falls upon its surface.^52^ Thus, the greater the value of *f*, the greater will be the LHE, and in return greater will be the photovoltaic abilities of the molecule.^53^ Between our newly reported series, the “a” series once again gave better results than its relative counterpart and thus could exhibit better charge transfer attributes than the “b” series. The values of LHE in Tables 4 and 5 were obtained from the formula written below.

7 LHE = 1 − 10^−*f*^

Here, LHE is the light harvesting ability, while *f* is the oscillator strength. Amongst all the reported molecules, IO1_a_ shows the highest values of oscillator strength and by association the highest LHE in both the gas phase and in the chloroform solvent.

### Solution processabilities

Dipole moment (*μ*) is a remarkable asset in the estimation of the solar efficiency of any photovoltaic cell. It has a direct association with the solubility of the molecule in the desired solvent. Molecules with high values of dipole moment are generally quite polar and thus are easily soluble in polar solvents.^54^ Though a symmetrical molecule should have a high *μ*, highly symmetrical molecules usually tend to have zero or close to zero dipole moment owing to the cancellation of opposite and antiparallel charges in them. A significant characteristic of dipole moment is that molecules with high values of dipole moment show better charge transfer between their FMOs.^55^ This should also be taken into account that molecules with low dipole moment, due to their highly symmetrical structure, have increased conjugation in them and thus could show better light absorption properties.^56^

Between the two newly reported series of designed molecules, when compared to the reference IOR, the “a” series shows a slight decrease, while the “b” series exhibits a significant increase in its dipole moment values. The lowered values of *μ* in the “a” series could be attributed to their planar topology. And the increased *μ* values in the “b” series could be due to their more twisted conformation. This could be one of the reasons behind the increase in the photovoltaic attributes of the molecules in the “a” series, and the better separation of HOMO–LUMO charge density in the “b” series. Table 6 represents the values of *μ* computed for both the gas (*μ*_g_) and the solvent (*μ*_s_) phase, along with their difference. The increased values of *μ*_s_ as compared to *μ*_g_ illustrate the stability of our newly formulated molecules in the chloroform solvent. The difference of only 0.000016 in the IO2_a_ molecule shows that this molecule could be effectively utilized in the active layer of OSCs irrespective of its medium. Overall, the highest dipole moment amongst all of IO2_b_ could be due to the twisted conformation, as well as the prominent electron transferring bridges present in this molecule, and implies its superior charge transfer features to all others.

**Table 6:** Dipole moment values in the gas (*μ*_g_) as well as the solvent (*μ*_s_) phase along with the difference between them (*μ*_s_–*μ*_g_)

Molecule	*μ*_g_ (Debye)	*μ*_s_ (Debye)	*μ*_s_–*μ*_g_ (Debye)
IOR	0.000616	0.000812	0.000196
IO1_a_	0.000510	0.000714	0.000204
IO2_a_	0.000316	0.000332	0.000016
IO3_a_	0.002500	0.003108	0.000608
IO1_b_	1.812156	2.379546	0.567390
IO2_b_	5.031879	6.825028	1.793149
IO3_b_	3.558277	4.845889	1.287612

### Molecular electrostatic potential (MESP) surfaces

MESPs are the hued contours around the molecules, which are attained through the selected functional and basis set at their ground state. These MESPs represent the potential a positive charge may experience when it is brought near the scrutinized molecule. These surfaces help to determine the effect of the structure on the reactivity, as well as the charge-density of the molecule.^51^ They also illustrate the sites of attack of the attacking reagent. These three-dimensional colored clouds around the molecules show a range of colors between two extremities of red and blue, where blue shows the maximum potential and red signifies the minimum potential.^57^ The red-colored areas are electron-deficient and thus are effective sites for the attack of an incoming nucleophile, while the blue-colored zones due to them being electron-rich acts as the sites of attack of an electrophile. The green-colored areas in the MESPs demonstrate the neutral or zero potential areas.^58^

**Fig. 6:**
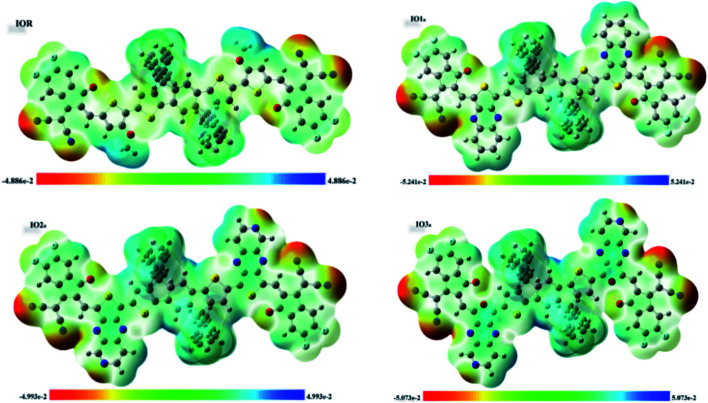
MESPs of IOR and “a” series of all the newly formulated molecules.

In the scrutinized molecules the red contours are seen all around the highly electronegative unsaturated oxygen and nitrogen atoms, while all the phenyl rings owing to them being electron-rich show blue-colored clouds around them (Fig. 6 (for reference and “a” series) and Fig. S6† (for “b” series)). The molecules of the “a” series show quite distinctive blue zones, while the red color is dispersed around the whole molecule in their counterparts of the “b” series. This provides us a shred of evidence towards our assumption that the molecules of the “a” series could act as better donor molecules and could have better charge transfer ability than that of the “b” series, accredited to the distinctive separation of charge density in these molecules.

### Exciton dissociation energy

As the name indicates it is the amount of energy needed in order to dismantle the short-lived excitons (electron–hole pairs) generated after the absorption of electromagnetic radiations.^59^ It is also known as the binding energy (*E*_b_), as it is the energy required to disintegrate the bound electron–hole pair. It has a direct relation with the coulombic interactions within the excitons. The reason behind the need to rapidly separate these charges is so that they can quickly and effectively move towards their respective electrodes and not recombine, and this way maximum photocurrent could be generated. So, the lower this binding energy is, the better will be the charge dissociation. *E*_b_ values in Table S3† are actually calculated through the difference between the values of optical and electronic band gap, as represented through the eqn (8) below^60,61^

8 *E*_b_ = *E*_g_ − *E*_x_

In the above equation, *E*_g_ is the electronic band gap, *E*_x_ demonstrates the optical band gap, while *E*_b_ shows the binding or exciton dissociation energy.

In gas phase, the newly reported molecules along with the reference IOR shows the decreasing order of IO1_b_ > IO3_b_ > IO2_b_ > IO3_a_ > IOR > IO1_a_ > IO2_a_, and a different trend of IO1_b_ > IO3_b_ > IOR > IO2_b_ > IO2_a_ > IO3_a_ > IO1_a_ is followed by them in the solvent phase. It is clear from these trends that the binding energy is the lowest for molecules of “a” series amongst all, with the slight exception of IO3_a_ in the gas phase. The lowest value of binding energy for IO2_a_ in the gas phase illustrates the improved capability of this molecule to dissociate the excitons in the gas phase, amongst all other studied molecules. Similarly, in the solvent phase, the lowest value of binding energy of IO1_a_, amongst all, shows its notable ability to transfer charges towards the separated electrodes in the solvent phase. The highest value of binding energy in both the proposed series was seen to be of IO3 (a and b), which could be due to the presence of highly electron withdrawing oxygen atom present in the bridges of this molecule that instead of transfer the charges between the donor and acceptor must have shifted the charges towards themselves, thus raising the value of binding energy of the corresponding molecule. Overall, the lower values of binding energies for molecules of “a” series, signifies their ability to act as better light harvesting photovoltaic materials than the cited IOR molecule, as well as the molecules of the “b” series. Also, upon comparison between the binding energies in the gas phase and the solvent one, the high values of *E*_b_ in the solvent phase than the gas phase actually emphases' the notable association of the researched molecules with the solvent chloroform.

### Transition density matrix

From the plots of transition density matrix (TDM), presence of electronic delocalization and localization in a molecule can be identified. From these plots, the excitation of electrons along with the position of excitons (electron–hole pair) can be estimated, as well.^62^ Moreover, the short-circuit current (*J*_SC_) in an organic photovoltaic cell can be evaluated through the analysis of charge density illustrated through the TDM plots. As the molecules having low electron–hole coupling (localization) tends to have high charge transfer rate, and thus high values of *J*_SC_.^63^

**Fig. 7:**
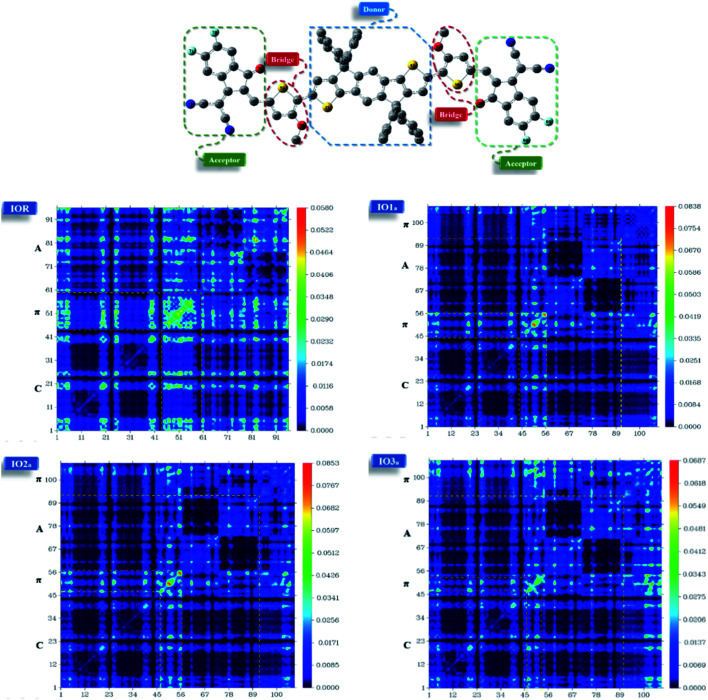
TDM plots of IOR and “a” series of all the newly designed molecules.

The TDM plots in Fig. 7 (for reference and “a” series) and Fig. S7† (for “b” series), were formulated using the MPW1PW91 functional of TD-DFT computations, at the 6-31G(d,p) basis set. The two-dimensional plots of TDM thus generated, have on their left *y*- and lower *x*-axis, the number of atoms present in the molecule except for hydrogen atoms. The reason behind the exclusion of hydrogen atoms (by default) is their insignificant contribution in the migration of charge density in the molecule.^64^ Along the right *y*-axis of the TDM plots, the charge density is represented with the help of different colors ranging from blue at one extreme to red at the other. The blue color demonstrates zero charge density, while red shows the maximum charge density present in the transition of the specific molecule.^65^ For easier evaluation, the number of atoms in the TDMs were fragmented into donor core (C), bridges (π), and acceptor (A) of the molecule. Here, between IO1_a_ and IO1_b_, the charge density is diagonally as well as off-diagonally spread over the bridges and acceptor components of the molecules, respectively, as represented by their brighter fringes in these areas as compared to others. But in case of all other designed molecules, the charge density is highly condensed over the π bridges. This spread of charge density signifies the contribution of the π-linkers in effective transfer of charges between acceptor and donor components of the molecules.

### Charge transfer mobilities

Another notably prominent tool in determining the charge transfer attributes of a molecule is to analyze its reorganization energies (*λ*). This *λ* can be evaluated using the semi-classical Marcus theory. This theory leads to the conclusion that charge transfer mobilities and reorganization energies (*λ*) have an inverse association between them. This claim can be justified through the eqn (9) below^66^9
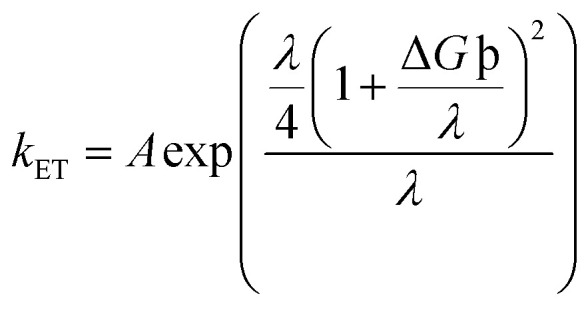


The *k*_ET_ in the above equation represents the rate of charge transfer and *A* directly depends on the type of charge transfer (whether intramolecular or intermolecular). The term Δ*G*° represents the standard free energy, which is close to zero in the self-exchange intramolecular transfer reaction studied for the newly derived molecules. Lastly, *λ* is the reorganization energy term and further consists of two parts; inner (*λ*_v_) and outer (*λ*_s_), as represented in eqn (10) below^66^

10 *λ* = *λ*_v_ + *λ*_s_

The latter directly corresponds to the external solvent response, and its effect is neglected in this study, due to the constant external chloroform environment. The former term (*λ*_v_) is related to the energy expenditure when a molecule switches its neutral geometry towards a charged geometry and *vice versa*.^67^ This *λ*_v_ further divides into electron (*λ*_e_) and hole (*λ*_h_) reorganization energies. Where *λ*_e_ is correlated to the energy utilized for the reorganization of the molecular geometry after the intramolecular transfer of electron, similar is the case for *λ*_h_ but with a hole instead of an electron transfer. The computed values for both the terms of *λ*_v_, *i.e.*, *λ*_e_ and *λ*_h_, were calculated with the help of eqn (1) and (2), and are represented in Table 7.

**Table 7:** Electron (*λ*_e_) and hole (*λ*_h_) reorganization energies values of all the researched molecules

Molecules	*λ*_h_ (hole)	*λ*_e_ (electron)
IOR	0.0077052	0.005041
IO1_a_	0.0060543	0.0067175
IO2_a_	0.0060808	0.0066025
IO3_a_	0.0063082	0.0065544
IO1_b_	0.0090177	0.0101874
IO2_b_	0.0090450	0.0106523
IO3_b_	0.0093598	0.0117805

Accredited to the lower number of electron withdrawing atoms (N and O) present in its bridges, the IO1_a_ molecule seemed to have higher hole mobility than other studied ones, as seen from its lowest *λ*_h_ value amongst all. In case of the reference molecule IOR, its lowest *λ*_e_ implies the notable ability of this molecule to act as the fullerene-free acceptor, as cited in many literatures.^68^ But in case of our proposed molecule, all of them, due to their lower values of *λ*_h_ relative to the *λ*_e_, could act as better donor molecules, as opposed to the reference molecule. Also, from the values of *λ*_h_, it is demonstrated that all the molecules of “a” series have lower *λ*_h_ than the cited molecule (IOR), while reverse is the case for the molecules of the “b” series. So, it can be evaluated that all the molecules of “a” series would have better transfer of hole within their molecular structure than that of the IOR and the other series of designed molecules. The higher values of both the evaluated reorganization energies for molecules of “b” series could be credited to the twisted confirmation of the molecules, which upon transfer of charges would require greater energy to reorganize themselves. On another note, though the values of *λ*_e_ are higher than IOR for both the “a” as well as “b” series, still the “a” series could show better electron mobilities than the “b” series due to their low *λ*_e_ values. Contrary to their counterparts, the lowered values of *λ*_h_ in Fig. S8,† provides a significant evidence towards our claim of the newly designed molecules being donors in the active layer of effective OSCs.

### Photo-conversion efficiency

The photo-conversion efficiency (*η*) of a molecule can be evaluated through the eqn (11) written below^69^11
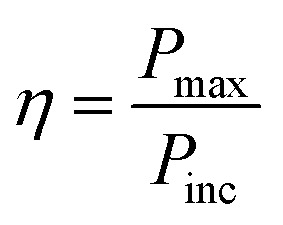


In the above equation, *P*_inc_ represents the intensity of radiant energy, which falls on the active layer of the organic photovoltaic cell, and *P*_max_ in the numerator can be computed through the eqn (12).^70^

12 *P*_max_ = *V*_OC_FF*J*_SC_

Here, the *V*_OC_, FF, and *J*_SC_ are the open-circuit voltage, fill factor, and short-circuit current, respectively, and can be calculated through the eqn (13), (14), and (15), sequentially.

The first term in the eqn (10) above, *i.e.*, the open-circuit voltage, is the maximum amount of voltage generated, when external supplied current is close to a minimum. This *V*_OC_ can be calculated through the eqn (13).^71^13
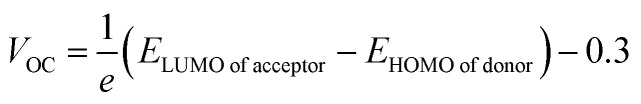
Where, *V*_OC_ is the open-circuit voltage, while 0.3 is an empirical factor. Moreover, *e* is the fundamental charge with a value of 1 in all the scrutinized molecules in this theoretical work. The values of open-circuit (*V*_OC_) along with the normalized *V*_OC_ (*v*_oc_) and fill factor are en-tabulated in Table 8. These values were acquired by taking all our investigated molecules as donor, and thus their HOMO values were then employed for evaluation of the *V*_OC_ by using the LUMO of PC_61_BM. PC_61_BM is a common and highly effective fullerene molecule, and is availed in many active layers as the acceptor component of the BHJ OSCs.

**Table 8:** Computed open-circuit voltage (*V*_OC_), normalized *V*_OC_ (*v*_oc_), and fill factor of the cited and the newly formulated molecules

Molecules	*V*_OC_ (V)	Normalized *V*_OC_ (*v*_oc_)	Fill factor
IOR	1.37	53.039	0.90776
IO1_a_	1.50	58.072	0.91410
IO2_a_	1.65	63.879	0.92161
IO3_a_	1.70	65.815	0.92220
IO1_b_	1.49	57.684	0.91365
IO2_b_	1.50	58.072	0.91410
IO3_b_	1.53	59.233	0.91544

**Fig. 8:**
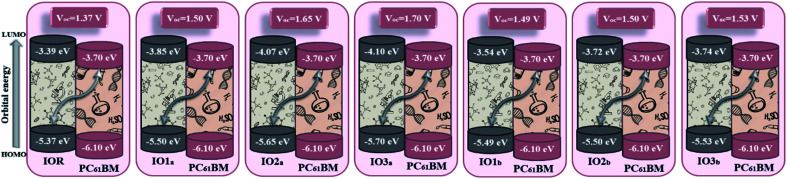
Open-circuit voltage of all the researched molecular donors attained with PC_61_BM acceptor molecule.

Upon comparison with the reference molecule IOR, all the newly presented molecules exhibited higher *V*_OC_, which signifies their better voltage generating ability (Fig. 8). While, between the molecules of “a” and “b” series, the molecules of the “a” series once again suppressed their twisted counterparts, owing to their larger *V*_OC_ than the “b” series molecules. While, within the “a” series, the highest open-circuit voltage was seen to be of IO3_a_, which could be accredited to its lowered and stabilized HOMO value.

The second evaluated factor for the PCE, the fill factor also has direct association with the photo-conversion efficiency of the molecule. This dimensionless value was determined through the eqn (14).14
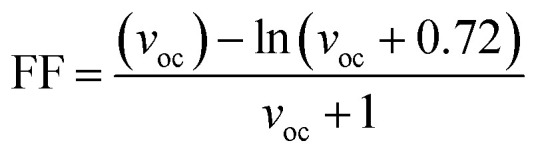


In the above equation, *v*_oc_ is the normalized *V*_OC_ and is enlisted in Table 8 using the formula 
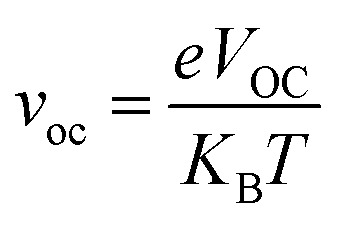
. Where, *e* is the charge of 1 on the molecule, *K*_B_ is the Boltzmann constant in eV and *T* is the average room temperature of 300 K.^61^ From the values of FF in Table 8, it can be evaluated that all the newly reported molecules can have better PCE than the reference molecule IOR, with the molecules of the “a” series being better than their alternates. Here, the IO3_a_ molecule, owing to its highest *V*_OC_, shows the maximum value of FF amongst all, and thus could be utilized in the active layer of organic solar cells with the aim of enhancing their solar efficiencies.

The last term in the numerator of eqn (12) is the short-circuit current (*J*_SC_) and can be theoretically calculated using the equation below^72^15
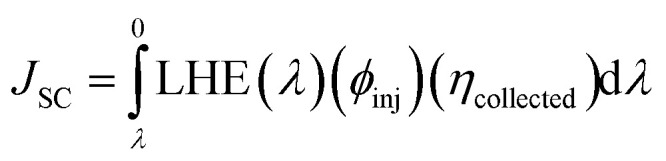
Here, the efficiency of injected electrons and that of charge collection are represented by *φ*_inj_ and *η*_collected_, sequentially,^73^ and the LHE can be equated using the eqn (7). Because of it being a theoretical work the value of short-circuit current couldn't be effectively calculated in this research work and for this reason we can just conclude that the higher this value is, the higher would be the PCE of the molecule in OSCs.

## Conclusion

To the study the effect of different π-linkers and their substitution positions on the opto-electronic properties of IOR molecule, various π-linkers were attached at two sites of attachment, *i.e.*, phenyl ring (b series) or thienyl/furyl ring (a series) of the same bridge. In this research work, three different bridges were substituted in the IOR molecule, and the thus formulated two series “a” and “b” were analyzed for their photo-physical and related photovoltaic attributes. It was seen that between these two series, the “a” series exhibited enhanced results in most of the studied properties. Such as, the maximum absorption values (*λ*_max_), excitons binding energy, electronic and optical band gap, open-circuit voltage, fill factor, chemical softness and hardness, and the hole reorganization energies, all gave outcomes in favor of utilizing the donor molecules of “a” series for the construction of effective organic photovoltaic cells. All these enhanced properties could be attributed to the extensively planar configuration of the molecules of the “a” series. The twisted molecules of the “b” series showed increased values of dipole moment along with better separation of charge densities in their FMOs. Though, the molecules of the “b” series were a step back from their superior counterparts, still they just like the molecules of the “a” series showed enhanced opto-electronic properties than the highly planar reference molecule IOR. This could be attributed to the presence of greater conjugation and enhanced charge transfer attributes in the newly substituted π-linkers in all the newly reported molecules than the ones in IOR. In addition, amongst all the proposed molecules of the “a” series, IO1_a_ and IO2_a_ seemed to show enhanced optoelectronic attributes, while IO3_a_ showed the highest values of factors related to the PCE. But, the values of these factors was also quite high for the other two molecules. So overall, we can conclude that both the π-linker and their substitution position directly affects the opto-electronic attributes of different photovoltaic chromophores. Also, all the newly reported molecules would be proficient substitutes to IOR in order to construct superiorly effective active layers in OSCs, with the “a” series being the best one.

## Conflicts of interest

The authors declare that they have no known competing financial interests or personal relationships that could have appeared to influence the work reported in this paper.

## Supplementary Material

RA-012-D2RA04097B-s001
