# Supplementary material for: Impact of various heterocyclic π-linkers and their substitution position on the opto-electronic attributes of the A–π–D–π–A type IECIO-4F molecule: a comparative analysis
Source: RSC Adv. 2022 Jul 20;12(32):20792–806. doi: 10.1039/d2ra04097b (PMC9297698; doi:10.1039/d2ra04097b)
Supplement: RA-012-D2RA04097B-s001 [file RA-012-D2RA04097B-s001.pdf]

Electronic Supplementary Material (ESI) for RSC Advances.
This journal is © The Royal Society of Chemistry 2022

SUPPORTING INFORMATION

Impact of various heterocyclic π-linkers and their substitution position on the opto-electronic attributes of the A-π-D-π-A type IECIO-4F molecule: A comparative analysis

Table S-1 Bond parameters of all the researched molecules

Molecules	Bond length "A_l" (Å)	Bond length "D_l" (Å)	Dihedral Angle (θ°_a)	Dihedral Angle (θ°_d)
**IOR**	1.41	1.43	0.01	0.41
**$IO1_a$**	1.40	1.42	0.00	0.01
**$IO2_a$**	1.40	1.42	0.00	0.02
**$IO3_a$**	1.39	1.41	0.27	0.04
**$IO1_b$**	1.44	1.45	41.2	21.8
**$IO2_b$**	1.46	1.45	44.7	4.72
**$IO3_b$**	1.46	1.45	45.8	19.0

Table: S-2. Various quantum chemical indices for the researched molecules.

Molecules	IP (eV)	EA (eV)	η (eV)	S (1/eV)
**IOR**	6.02	2.74	1.94	0.26
**$IO1_a$**	6.06	3.22	1.42	0.35
**$IO2_a$**	6.21	3.43	1.39	0.36
**$IO3_a$**	6.25	3.46	1.39	0.36
**$IO1_b$**	6.12	2.90	1.61	0.31
**$IO2_b$**	6.14	3.08	1.53	0.33
**$IO3_b$**	6.18	3.09	1.54	0.32

Table: S-3 Exciton dissociation energies of IOR along with the newly derived molecules in the both the evaluated phases.

**Molecules**	E_b **(eV) gaseous**	E_b **(eV) solvent**
**IOR**	0.2952	0.4176
**IO1$_a$**	0.2784	0.3702
**IO2$_a$**	0.2757	0.3790
**IO3$_a$**	0.2982	0.3771
**IO1$_b$**	0.3765	0.4425
**IO2$_b$**	0.3582	0.4108
**IO3$_b$**	0.3611	0.4265

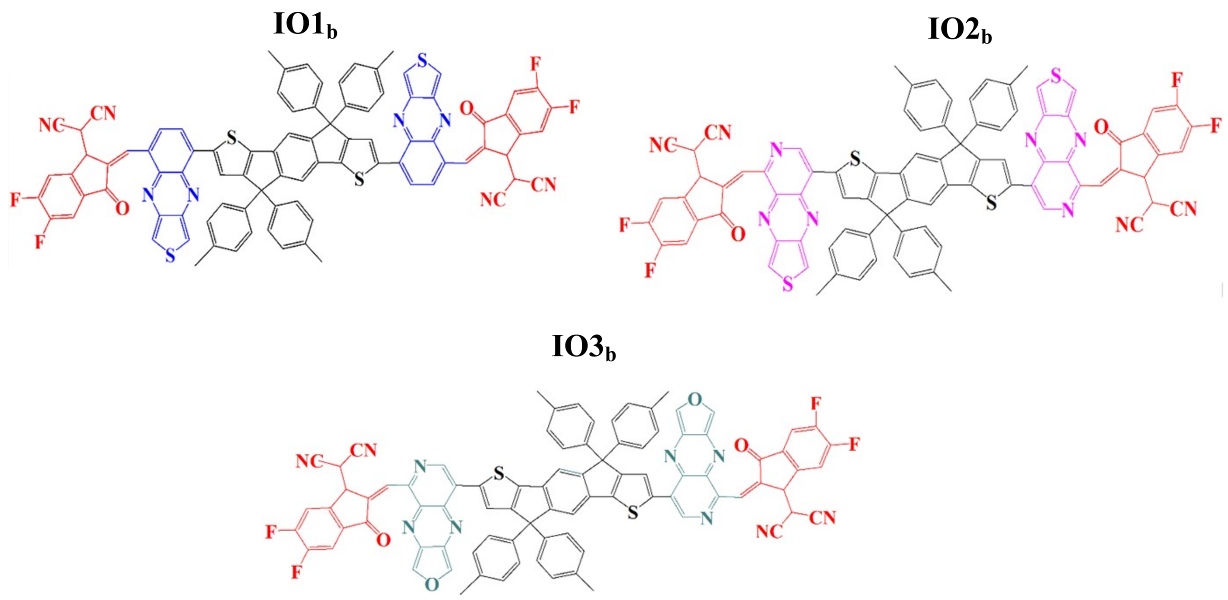

Fig.S-1. Pictorial representation of "b series" of designed molecules (Donor core is colored black, acceptors are red in colour, while blue, pink, and green represents various bridges)

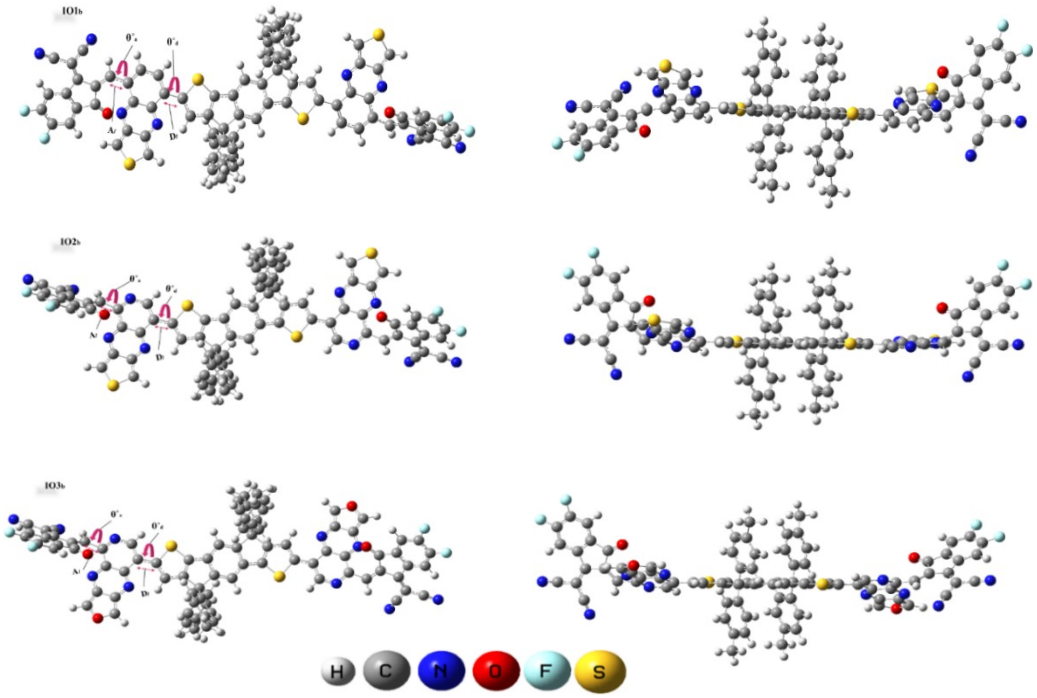

Fig.S-2. GaussView structures of "b" series of the newly reported molecules.

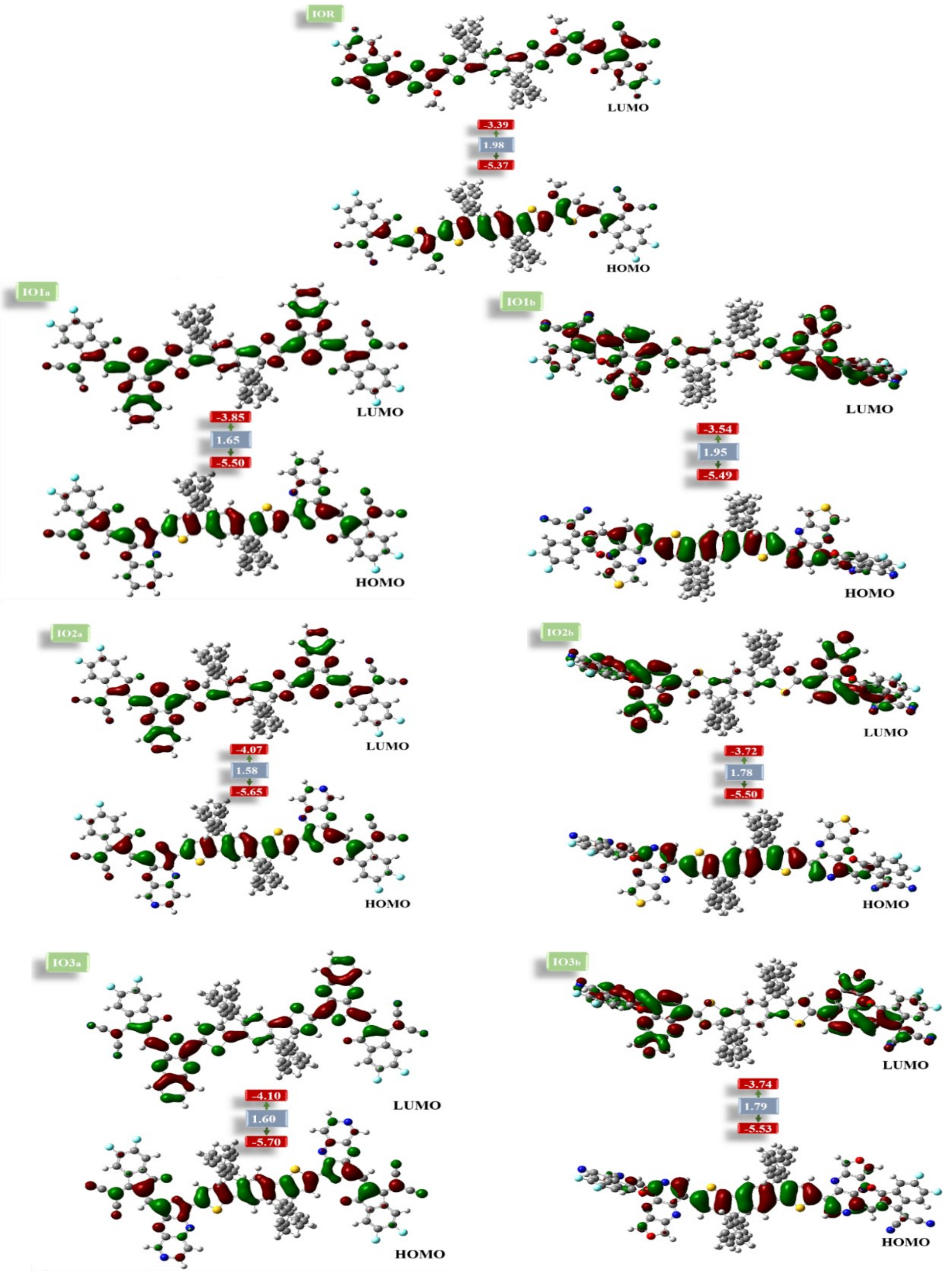

Fig.S-3. The studied FMOs of all the investigated molecules and the band gap between them.

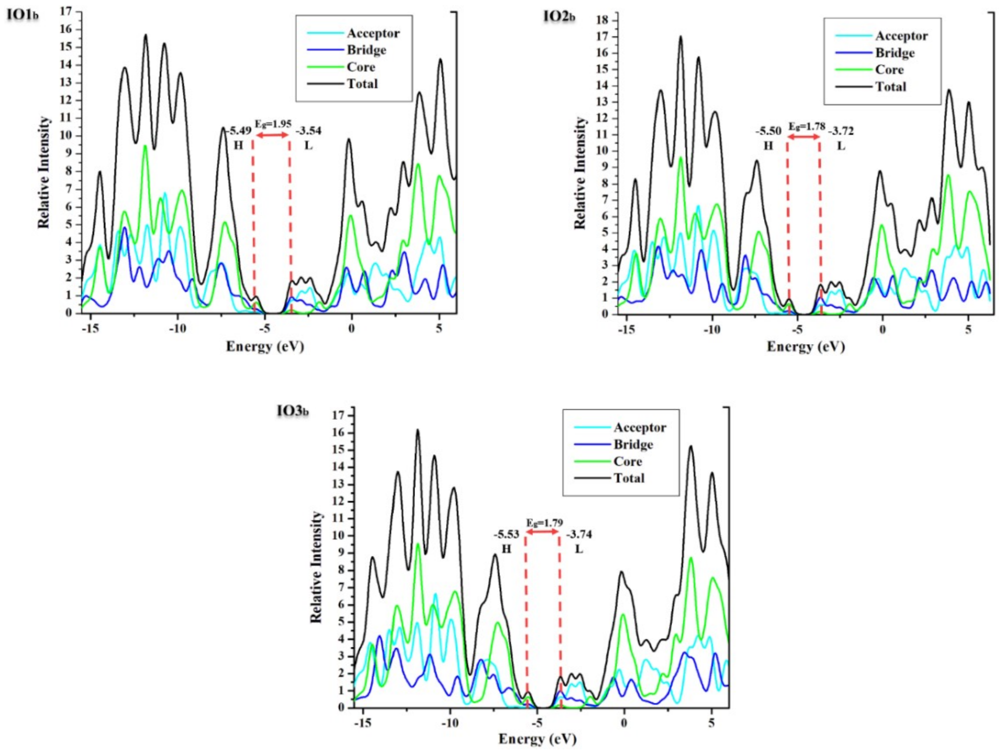

Fig.4. DOS graphs of “b” series of the newly reported molecules

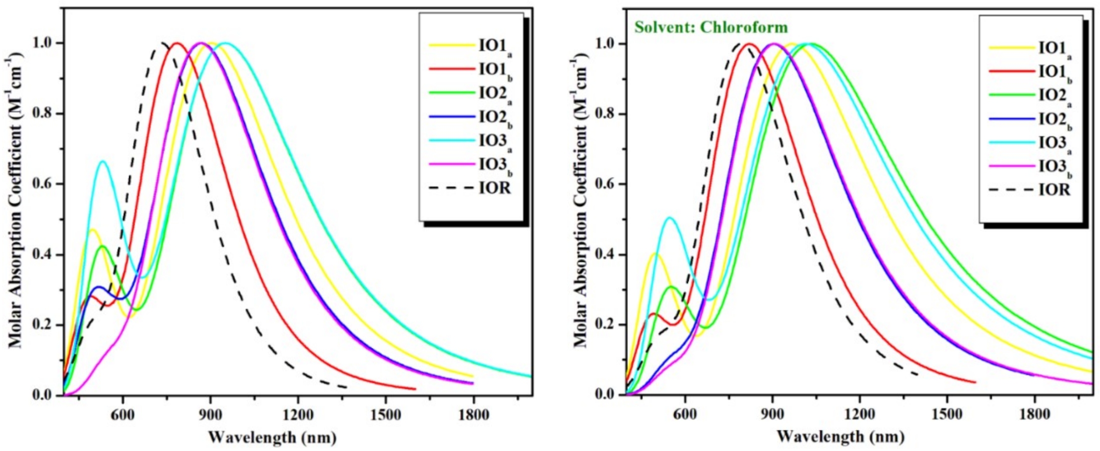

Fig.S-5. Absorption profile of IOR and all the reported molecules in gas (left) and solvent (right) phase

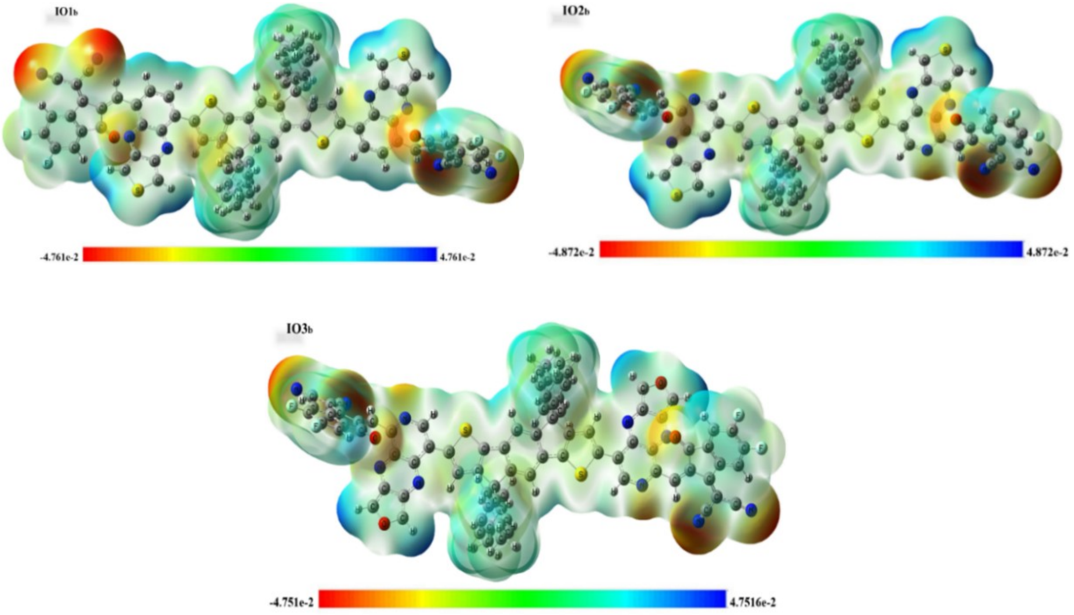

Fig.S-6. MESPs of "b" series of all the newly formulated molecules.

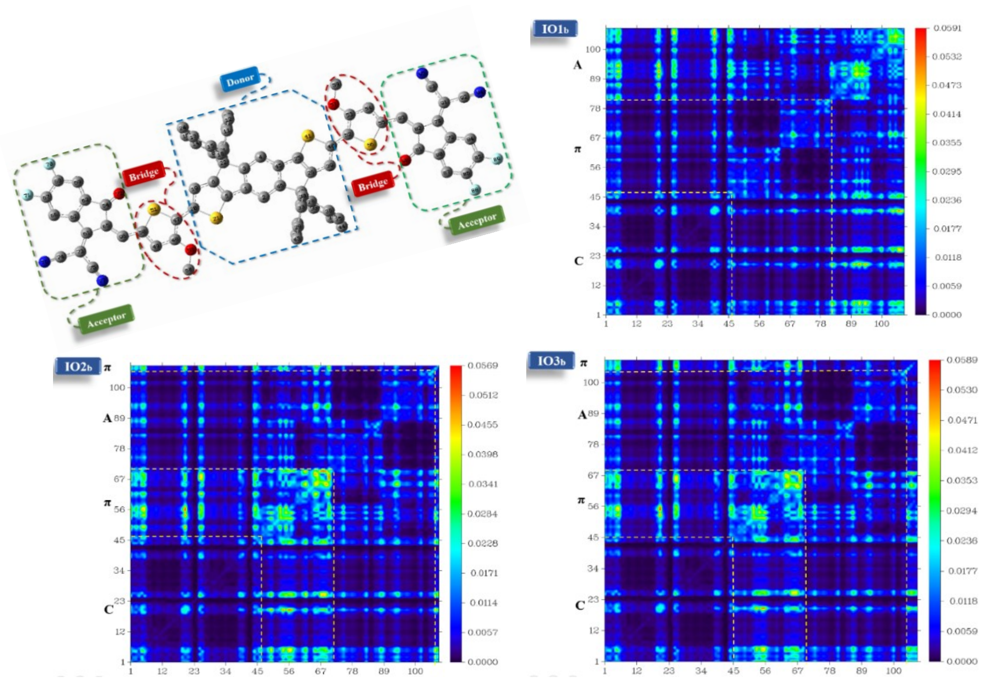

Fig.S-7. TDM plots of "b" series of all the newly designed molecules

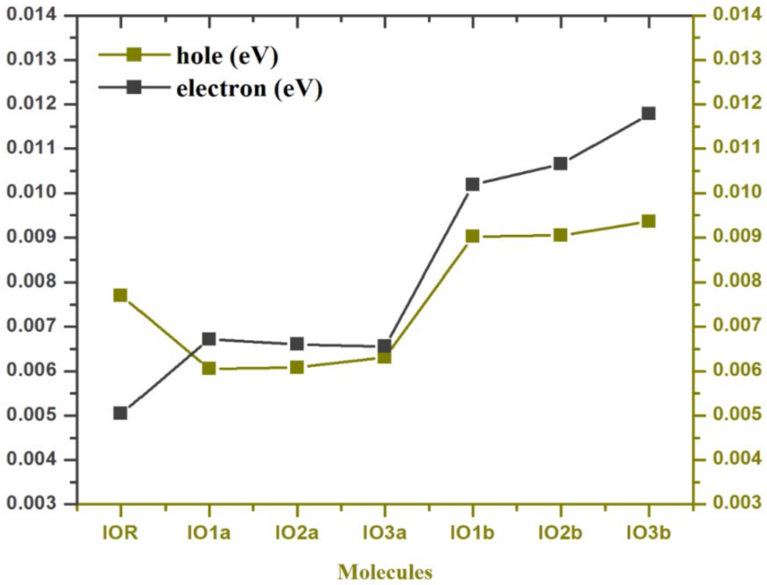

Fig.S-8. Graph illustrating the reorganization energies of both the hole and electron in all the investigated molecules

Table: S-4 Cartesian coordinates of reference (IOR) and designed molecules ($IO1_a$-$IO3_a$ and $IO1_b$-$IO3_b$) through MPW1PW91/6-31G (d,p) level of theory.

Cartesian coordinates of Reference molecule IOR at MPW1PW91/6-31G level of theory.

Center Number	Atomic Number	Atomic Type	Coordinates (Angstroms) X	Y	Z
1	6	0	-1.265859	0.526767	0.147938
2	6	0	-0.351608	-0.656897	0.242007
3	6	0	-0.909657	1.808047	-0.001550
4	6	0	0.497999	2.186608	-0.173742
5	6	0	-2.703948	0.500922	0.171097
6	6	0	-3.186762	1.688806	0.054111
7	6	0	-2.096863	3.544287	-1.284635
8	6	0	-2.294477	3.600501	1.246069
9	6	0	-1.956283	4.972412	-3.686061
10	6	0	-2.708319	3.040901	-2.440413
11	6	0	-1.452135	4.786805	-1.316008

12	6	0	-1.365448	5.492023	-2.524007
13	6	0	-2.648690	3.757665	-3.638819
14	1	0	-3.129355	3.376470	-4.516910
15	6	0	-3.177187	4.694740	1.203090
16	6	0	-1.778010	4.118272	3.559896
17	6	0	-3.394443	5.467337	2.353523
18	6	0	-2.691884	5.181395	3.531143
19	6	0	-1.596970	3.315146	2.424312
20	6	0	-4.631212	1.763064	0.075416
21	6	0	-5.063889	0.492588	0.149299
22	16	0	-3.834053	-0.760458	0.339070
23	6	0	-2.130590	2.710584	0.002513
24	6	0	-1.840737	5.736522	-5.015022
25	6	0	-2.925719	6.033604	4.793812
26	1	0	-2.781157	5.428902	5.664868
27	1	0	-2.232922	6.848514	4.806283
28	1	0	-3.925645	6.415593	4.787364
29	6	0	2.775367	1.131554	0.001311
30	6	0	3.220553	-0.116378	0.092797
31	6	0	2.108113	-2.025199	1.407798
32	6	0	2.307547	-2.079897	-1.123183
33	6	0	1.961566	-3.458157	3.804976
34	6	0	2.718633	-1.518782	2.565645
35	6	0	1.463633	-3.261917	1.434997
36	6	0	1.372542	-3.972183	2.640209
37	6	0	2.654885	-2.242445	3.761125
38	1	0	3.132025	-1.865157	4.641330
39	6	0	1.599602	-1.794032	-2.297538
40	6	0	3.400036	-3.942146	-2.235224
41	6	0	1.769261	-2.601575	-3.431340
42	6	0	2.685987	-3.661678	-3.407671
43	6	0	3.192934	-3.166666	-1.085668
44	6	0	4.713931	-0.208416	0.042676
45	6	0	5.228832	1.055889	-0.000748

46	16	0	4.002730	2.339135	-0.026417
47	6	0	2.137541	-1.191799	0.122193
48	6	0	1.845949	-4.226921	5.134264
49	6	0	2.909564	-4.517458	-4.670094
50	1	0	3.910431	-4.897363	-4.670648
51	1	0	2.218522	-5.333577	-4.676705
52	1	0	2.757538	-3.913878	-5.540909
53	6	0	1.317630	1.150117	-0.058996
54	6	0	0.898359	-0.325695	0.103233
55	1	0	1.196980	-2.410019	-4.314587
56	1	0	0.927706	-0.961193	-2.329204
57	1	0	4.102704	-4.749024	-2.217257
58	1	0	3.718368	-3.398293	-0.183096
59	1	0	0.848750	-4.904303	2.673204
60	1	0	3.231808	-0.580666	2.536405
61	1	0	-0.845975	6.425202	-2.560064
62	1	0	-1.022818	5.190793	-0.424817
63	1	0	-1.220813	3.916711	4.450199
64	1	0	-3.694789	4.931710	0.296858
65	1	0	-4.100863	6.271555	2.333849
66	1	0	-0.928177	6.294913	-5.025529
67	1	0	-1.842042	5.040187	-5.828015
68	1	0	-2.669553	6.404147	-5.115781
69	1	0	1.031414	-3.661387	0.540397
70	1	0	-0.700614	-1.644202	0.397020
71	1	0	0.934086	-4.786569	5.144387
72	1	0	2.675678	-4.894489	5.233227
73	1	0	1.847346	-3.532858	5.949310
74	1	0	-5.208243	2.655621	0.044551
75	1	0	-0.926009	2.483316	2.457663
76	1	0	-3.224032	2.103217	-2.404349
77	1	0	0.811178	3.173638	-0.350676
78	1	0	5.273923	-1.117108	0.044841
79	6	0	-6.408972	0.052930	0.113095

80	6	0	-6.631672	-1.257469	0.246509
81	6	0	-8.015914	-1.516004	0.316908
82	6	0	-8.758036	-0.457731	0.119674
83	16	0	-7.880210	1.048117	0.002091
84	1	0	-8.401234	-2.496599	0.500177
85	6	0	6.670633	1.397367	0.036315
86	6	0	7.106499	2.673575	0.039570
87	6	0	8.608391	2.741065	-0.026756
88	6	0	9.179316	1.513576	-0.048423
89	16	0	7.985537	0.229922	0.110935
90	1	0	9.155281	3.661438	-0.062095
91	8	0	6.279077	3.840355	0.089795
92	8	0	-5.788752	-2.392502	0.326413
93	6	0	7.065097	5.002725	-0.273074
94	6	0	-6.764124	-3.472628	0.114442
95	1	0	-7.508614	-3.441202	0.895100
96	1	0	-7.253203	-3.337627	-0.833174
97	1	0	-6.281808	-4.424234	0.130275
98	1	0	6.428584	5.858179	-0.316519
99	1	0	7.833473	5.167412	0.462092
100	1	0	7.526922	4.850192	-1.228280
101	6	0	-12.125359	-1.727785	-0.211270
102	6	0	-13.490016	-1.451050	-0.221906
103	6	0	-13.677698	0.034347	-0.024589
104	6	0	-12.280902	0.711498	0.093538
105	6	0	-11.333827	-0.517166	-0.005884
106	6	0	-14.419266	-2.348766	-0.353917
107	6	0	-15.838916	-1.871599	-0.251560
108	6	0	-16.088632	-0.537471	-0.099862
109	6	0	-14.930515	0.502307	0.017797
110	1	0	-14.170564	-3.368461	-0.512014
111	1	0	-15.138316	1.531215	0.133406
112	9	0	-17.333765	-0.100489	-0.053147
113	9	0	-16.800042	-2.786750	-0.315243

114	8	0	-12.031659	1.915867	0.249976
115	6	0	-11.643098	-2.895057	-0.348566
116	6	0	-12.570278	-4.051026	-0.630972
117	6	0	-10.138835	-3.179009	-0.232445
118	7	0	-13.255627	-4.905386	-0.839704
119	7	0	-9.038168	-3.454849	-0.153154
120	6	0	12.787787	0.019566	-0.138432
121	6	0	13.136846	-1.419271	-0.071660
122	6	0	12.027656	-2.147991	-0.013666
123	6	0	10.781077	-1.260024	-0.035997
124	6	0	11.306366	0.162486	-0.128652
125	6	0	10.674969	1.344256	-0.183508
126	6	0	14.528223	-2.034297	-0.064117
127	6	0	14.613648	-3.384741	-0.001516
128	6	0	13.327939	-4.248500	0.065174
129	6	0	12.098225	-3.665781	0.062484
130	1	0	15.396805	-1.431208	-0.107133
131	1	0	11.217063	-4.246806	0.111116
132	9	0	13.446828	-5.574331	0.125179
133	9	0	15.806545	-3.988437	0.003882
134	8	0	9.592085	-1.677198	0.014383
135	6	0	13.650373	1.023861	-0.190544
136	6	0	15.171162	0.784271	-0.201000
137	6	0	13.121513	2.453591	-0.233707
138	7	0	16.302061	0.686824	-0.211291
139	7	0	12.780683	3.533339	-0.271394
140	1	0	11.250127	2.222778	-0.319015
141	6	0	-10.104952	-0.932912	0.019270
142	1	0	-10.075682	-2.001311	-0.034688

Cartesian coordinates of designed molecule IO1$_a$ at MPW1PW91/6-31G level of theory.

Center Number	Atomic Number	Atomic Type	Coordinates (Angstroms) X	Y	Z
1	6	0	1.275695	0.409403	0.052832

2	6	0	0.286939	1.404631	0.100176
3	6	0	0.948363	-0.960643	0.009924
4	6	0	-0.339409	-1.401689	-0.073580
5	6	0	2.683122	0.538035	-0.010728
6	6	0	3.294586	-0.647975	-0.038245
7	6	0	2.230407	-2.717155	-1.203249
8	6	0	2.473014	-2.592431	1.326557
9	6	0	2.104804	-4.321001	-3.501772
10	6	0	2.767597	-2.259091	-2.411404
11	6	0	1.658937	-3.998666	-1.132385
12	6	0	1.583987	-4.794436	-2.288073
13	6	0	2.709087	-3.059262	-3.558327
14	1	0	3.124180	-2.704683	-4.478695
15	6	0	3.396254	-3.647606	1.344521
16	6	0	1.996990	-2.983376	3.681227
17	6	0	3.653593	-4.338203	2.538492
18	6	0	2.953854	-4.007346	3.706509
19	6	0	1.764243	-2.267877	2.495429
20	6	0	4.720189	-0.598815	-0.020306
21	6	0	5.204255	0.762409	-0.039401
22	16	0	3.820006	1.892615	-0.053805
23	6	0	2.252422	-1.784906	0.025896
24	6	0	2.013564	-5.184854	-4.774222
25	6	0	3.237886	-4.768144	5.017024
26	1	0	3.037615	-4.128724	5.851697
27	1	0	2.609953	-5.632552	5.071326
28	1	0	4.264369	-5.070994	5.038616
29	6	0	-2.728564	-0.537437	-0.024083
30	6	0	-3.345601	0.644802	0.019027
31	6	0	-2.304278	2.715059	1.207837
32	6	0	-2.537763	2.594385	-1.323517
33	6	0	-2.181605	4.324081	3.503258
34	6	0	-2.845918	2.259211	2.416499
35	6	0	-1.737825	3.997441	1.134739
36	6	0	-1.662456	4.795347	2.288266

37	6	0	-2.787014	3.062662	3.562202
38	1	0	-3.202511	2.711191	4.483386
39	6	0	-1.831833	2.271583	-2.493019
40	6	0	-3.725027	4.339517	-2.535145
41	6	0	-2.067259	2.987187	-3.678423
42	6	0	-3.025329	4.010178	-3.703706
43	6	0	-3.466355	3.647567	-1.341204
44	6	0	-4.764259	0.594594	0.001223
45	6	0	-5.237500	-0.753116	0.009840
46	16	0	-3.858618	-1.890305	-0.101065
47	6	0	-2.312981	1.784977	-0.023471
48	6	0	-2.087137	5.189838	4.774785
49	6	0	-3.309692	4.771693	-5.014717
50	1	0	-4.336241	5.074611	-5.036445
51	1	0	-2.681818	5.636245	-5.068829
52	1	0	-3.109375	4.132573	-5.849665
53	6	0	-1.324948	-0.405701	-0.044171
54	6	0	-1.001426	0.964073	0.005917
55	1	0	-1.516425	2.750213	-4.564915
56	1	0	-1.112424	1.480082	-2.481946
57	1	0	-4.458505	5.118981	-2.553997
58	1	0	-3.985686	3.917587	-0.445747
59	1	0	-1.206582	5.762584	2.241734
60	1	0	-3.303163	1.293318	2.464719
61	1	0	1.127200	-5.761557	-2.244469
62	1	0	1.272210	-4.366104	-0.204381
63	1	0	1.445351	-2.746089	4.567373
64	1	0	3.912202	-3.920446	0.448512
65	1	0	4.386123	-5.118201	2.557626
66	1	0	1.135690	-5.795157	-4.729282
67	1	0	1.961838	-4.549196	-5.634153
68	1	0	2.879173	-5.809185	-4.843964
69	1	0	-1.355090	4.363572	0.205072
70	1	0	0.530687	2.441121	0.187651
71	1	0	-1.208610	5.799244	4.727697

72	1	0	-2.951873	5.815355	4.845450
73	1	0	-2.034578	4.555130	5.635357
74	1	0	5.347897	-1.467975	0.005473
75	1	0	1.044348	-1.476434	2.483171
76	1	0	3.221595	-1.292320	-2.457384
77	1	0	-0.586423	-2.441664	-0.144337
78	1	0	-5.398193	1.456276	-0.010120
79	6	0	6.582902	1.159625	-0.097298
80	6	0	7.127784	2.511081	-0.081631
81	6	0	8.537204	2.542236	-0.107696
82	6	0	9.148400	1.293542	-0.105165
83	16	0	7.936837	0.001314	-0.216389
84	6	0	-6.599361	-1.137257	0.109586
85	6	0	-7.074656	-2.473722	0.088398
86	6	0	-8.479138	-2.565778	0.087634
87	6	0	-9.159069	-1.331237	0.112559
88	16	0	-7.979025	-0.015812	0.279821
89	6	0	12.828167	0.097947	-0.028788
90	6	0	13.294788	-1.365441	-0.025528
91	6	0	12.200322	-2.217288	-0.026091
92	6	0	10.879879	-1.396616	-0.029518
93	6	0	11.294493	0.082389	-0.025529
94	6	0	14.594538	-1.848902	-0.026278
95	6	0	14.762356	-3.198845	-0.024966
96	6	0	13.632748	-4.063556	-0.023905
97	6	0	12.359547	-3.558485	-0.025099
98	1	0	15.433857	-1.185644	-0.027885
99	1	0	11.511309	-4.212484	-0.024878
100	9	0	13.821253	-5.399019	-0.021522
101	9	0	16.008364	-3.720045	-0.024318
102	8	0	9.707932	-1.857654	-0.029374
103	6	0	13.618423	1.209097	-0.034555
104	6	0	15.011843	1.086713	-0.038198
105	6	0	13.040555	2.482841	-0.043818
106	7	0	16.155182	1.004169	-0.039605

107	7	0	12.577641	3.534988	-0.051488
108	6	0	-12.876753	-0.122266	0.029910
109	6	0	-13.342873	1.344470	0.029689
110	6	0	-12.247746	2.195480	0.031733
111	6	0	-10.927294	1.368540	0.035723
112	6	0	-11.336276	-0.119122	0.026533
113	6	0	-10.574729	-1.260350	0.018693
114	6	0	-14.646579	1.834013	0.029090
115	6	0	-14.815101	3.185525	0.027216
116	6	0	-13.685839	4.047935	0.026849
117	6	0	-12.410439	3.540493	0.029971
118	1	0	-15.488401	1.173605	0.030203
119	1	0	-11.564914	4.196244	0.030722
120	9	0	-13.875149	5.385298	0.023387
121	9	0	-16.061834	3.708407	0.025679
122	8	0	-9.755649	1.829658	0.042956
123	6	0	-13.677187	-1.222769	0.036925
124	6	0	-15.068989	-1.084327	0.039251
125	6	0	-13.111306	-2.501354	0.045334
126	7	0	-16.211569	-0.986519	0.040728
127	7	0	-12.647478	-3.550858	0.052233
128	1	0	-11.099008	-2.188675	-0.059674
129	6	0	10.549844	1.214710	-0.025247
130	1	0	11.081391	2.138009	0.039454
131	6	0	-8.346090	-4.974181	0.035376
132	6	0	-6.935348	-4.902323	0.037819
133	6	0	-8.990641	-6.218294	0.014904
134	6	0	-8.241451	-7.399442	0.004077
135	6	0	-6.841663	-7.339978	0.006969
136	6	0	-6.186542	-6.094970	0.020907
137	1	0	-10.059769	-6.264381	0.008603
138	1	0	-8.738879	-8.347719	-0.006499
139	1	0	-6.271666	-8.246355	-0.001096
140	1	0	-5.117287	-6.058002	0.018856
141	6	0	8.609479	4.960306	-0.052013

142	6	0	7.193776	4.979762	-0.017233
143	6	0	8.690045	7.400666	0.048171
144	6	0	7.291281	7.440911	0.061298
145	6	0	6.541288	6.242932	0.032277
146	6	0	9.347617	6.164389	-0.004209
147	1	0	9.257368	8.314406	0.082482
148	1	0	6.793474	8.394311	0.087963
149	1	0	5.477042	6.301670	0.055741
150	1	0	10.421593	6.137500	-0.008919
151	7	0	-6.287057	-3.640569	0.058424
152	7	0	-9.118403	-3.803694	0.053827
153	7	0	6.437573	3.752154	-0.033560
154	7	0	9.274813	3.733135	-0.115593

Cartesian coordinates of designed molecule IO2$_a$ at MPW1PW91/6-31G level of theory.

Center Number	Atomic Number	Atomic Type	Coordinates (Angstroms) X	Y	Z
1	6	0	1.275579	0.410000	0.052899
2	6	0	0.286320	1.404724	0.100296
3	6	0	0.948941	-0.960209	0.009903
4	6	0	-0.338607	-1.401901	-0.073638
5	6	0	2.682942	0.539347	-0.010642
6	6	0	3.295006	-0.646351	-0.038228
7	6	0	2.231883	-2.715997	-1.203367
8	6	0	2.474407	-2.591307	1.326449
9	6	0	2.107110	-4.319766	-3.501990
10	6	0	2.768851	-2.257587	-2.411490
11	6	0	1.661061	-3.997802	-1.132586
12	6	0	1.586522	-4.793539	-2.288324
13	6	0	2.710754	-3.057718	-3.558462
14	1	0	3.125676	-2.702872	-4.478805
15	6	0	3.398180	-3.646016	1.344355
16	6	0	1.998562	-2.982637	3.681090
17	6	0	3.655859	-4.336555	2.538285
18	6	0	2.955943	-4.006124	3.706317

19	6	0	1.765462	-2.267184	2.495334
20	6	0	4.720584	-0.596472	-0.020275
21	6	0	5.203961	0.764998	-0.039282
22	16	0	3.819140	1.894505	-0.053627
23	6	0	2.253417	-1.783813	0.025835
24	6	0	2.016316	-5.183586	-4.774493
25	6	0	3.240351	-4.766859	5.016788
26	1	0	3.039750	-4.127592	5.851498
27	1	0	2.612854	-5.631588	5.071031
28	1	0	4.266986	-5.069192	5.038370
29	6	0	-2.728199	-0.538861	-0.024106
30	6	0	-3.345835	0.643063	0.019072
31	6	0	-2.305568	2.713773	1.208017
32	6	0	-2.538972	2.593137	-1.323346
33	6	0	-2.183727	4.322716	3.503537
34	6	0	-2.846987	2.257577	2.416647
35	6	0	-1.739764	3.996447	1.135003
36	6	0	-1.664808	4.794319	2.288579
37	6	0	-2.788498	3.060987	3.562399
38	1	0	-3.203825	2.709250	4.483559
39	6	0	-1.832870	2.270763	-2.492862
40	6	0	-3.727109	4.337742	-2.534876
41	6	0	-2.068649	2.986322	-3.678225
42	6	0	-3.027236	4.008829	-3.703453
43	6	0	-3.468097	3.645850	-1.340976
44	6	0	-4.764467	0.592138	0.001254
45	6	0	-5.237026	-0.755812	0.009784
46	16	0	-3.857568	-1.892296	-0.101181
47	6	0	-2.313791	1.783763	-0.023348
48	6	0	-2.089708	5.188442	4.775118
49	6	0	-3.311974	4.770281	-5.014419
50	1	0	-4.338675	5.072681	-5.036136
51	1	0	-2.684537	5.635154	-5.068472
52	1	0	-3.111327	4.131313	-5.849404
53	6	0	-1.324650	-0.406414	-0.044175

54	6	0	-1.001822	0.963521	0.006000
55	1	0	-1.517688	2.749680	-4.564727
56	1	0	-1.113061	1.479627	-2.481832
57	1	0	-4.460982	5.116837	-2.553686
58	1	0	-3.987571	3.915552	-0.445506
59	1	0	-1.209422	5.761790	2.242110
60	1	0	-3.303744	1.291450	2.464804
61	1	0	1.130225	-5.760894	-2.244783
62	1	0	1.274512	-4.365493	-0.204608
63	1	0	1.446796	-2.745684	4.567247
64	1	0	3.914273	-3.918540	0.448333
65	1	0	4.388784	-5.116184	2.557377
66	1	0	1.138751	-5.794337	-4.729598
67	1	0	1.964276	-4.547902	-5.634386
68	1	0	2.882242	-5.807475	-4.844267
69	1	0	-1.357207	4.362828	0.205361
70	1	0	0.529543	2.441332	0.187836
71	1	0	-1.211488	5.798296	4.728075
72	1	0	-2.954760	5.813517	4.845816
73	1	0	-2.036834	4.553709	5.635652
74	1	0	5.348731	-1.465315	0.005456
75	1	0	1.045167	-1.476104	2.483120
76	1	0	3.222360	-1.290584	-2.457406
77	1	0	-0.585095	-2.441997	-0.144460
78	1	0	-5.398837	1.453500	-0.010042
79	6	0	6.582407	1.162915	-0.097143
80	6	0	7.126606	2.514646	-0.081389
81	6	0	8.536010	2.546515	-0.107441
82	6	0	9.147837	1.298131	-0.104982
83	16	0	7.936929	0.005296	-0.216295
84	6	0	-6.598694	-1.140647	0.109496
85	6	0	-7.073313	-2.477351	0.088222
86	6	0	-8.477747	-2.570118	0.087441
87	6	0	-9.158303	-1.335923	0.112436
88	16	0	-7.978926	-0.019911	0.279788

89	6	0	12.828208	0.104392	-0.028649
90	6	0	13.295570	-1.358759	-0.025476
91	6	0	12.201535	-2.211160	-0.026100
92	6	0	10.880677	-1.391155	-0.029486
93	6	0	11.294542	0.088058	-0.025403
94	6	0	14.595564	-1.841563	-0.026245
95	6	0	14.764065	-3.191421	-0.025014
96	6	0	13.634894	-4.056704	-0.024016
97	6	0	12.361438	-3.552276	-0.025189
98	1	0	15.434548	-1.177880	-0.027805
99	1	0	11.513531	-4.206704	-0.025015
100	9	0	13.824074	-5.392071	-0.021714
101	9	0	16.010336	-3.711990	-0.024389
102	8	0	9.708963	-1.852786	-0.029381
103	6	0	13.617902	1.215943	-0.034342
104	6	0	15.011384	1.094263	-0.037982
105	6	0	13.039390	2.489394	-0.043532
106	7	0	16.154764	1.012299	-0.039385
107	7	0	12.575944	3.541308	-0.051140
108	6	0	-12.876598	-0.128827	0.029832
109	6	0	-13.343459	1.337673	0.029698
110	6	0	-12.248763	2.189237	0.031803
111	6	0	-10.927893	1.362964	0.035752
112	6	0	-11.336122	-0.124904	0.026468
113	6	0	-10.573998	-1.265745	0.018563
114	6	0	-14.647413	1.826556	0.029118
115	6	0	-14.816618	3.177983	0.027326
116	6	0	-13.687793	4.040964	0.027022
117	6	0	-12.412136	3.534168	0.030122
118	1	0	-15.488900	1.165723	0.030184
119	1	0	-11.566943	4.190345	0.030920
120	9	0	-13.877780	5.378232	0.023640
121	9	0	-16.063616	3.700235	0.025812
122	8	0	-9.756481	1.824674	0.043023
123	6	0	-13.676475	-1.229736	0.036774

124	6	0	-15.068347	-1.091998	0.039097
125	6	0	-13.109947	-2.508034	0.045108
126	7	0	-16.210976	-0.994768	0.040570
127	7	0	-12.645588	-3.557304	0.051947
128	1	0	-11.097807	-2.194332	-0.059864
129	6	0	10.549320	1.220002	-0.025058
130	1	0	11.080400	2.143567	0.039705
131	6	0	-8.343480	-4.978450	0.035036
132	6	0	-6.932775	-4.905879	0.037495
133	6	0	-8.987402	-6.222888	0.014482
134	6	0	-6.837857	-7.343484	0.006495
135	6	0	-6.183366	-6.098146	0.020515
136	1	0	-10.056507	-6.269515	0.008170
137	1	0	-6.267401	-8.249572	-0.001621
138	1	0	-5.114130	-6.060637	0.018474
139	6	0	8.607062	4.964618	-0.051609
140	6	0	7.191348	4.983356	-0.016839
141	6	0	7.287607	7.444549	0.061845
142	6	0	6.538222	6.246193	0.032744
143	6	0	9.344590	6.169071	-0.003725
144	1	0	6.789319	8.397696	0.088564
145	1	0	5.473946	6.304391	0.056202
146	1	0	10.418579	6.142727	-0.008428
147	7	0	-6.285123	-3.643798	0.058182
148	7	0	-9.116386	-3.808355	0.053553
149	7	0	6.435767	3.755367	-0.033247
150	7	0	9.273016	3.737788	-0.115260
151	7	0	-8.237614	-7.403655	0.003589
152	7	0	8.686392	7.405012	0.048725

Cartesian coordinates of designed molecule $IO3_a$ at MPW1PW91/6-31G level of theory.

Center Number	Atomic Number	Atomic Type	Coordinates (Angstroms) X	Y	Z
1	6	0	1.275143	0.409886	0.052840
2	6	0	0.285878	1.404577	0.100803

3	6	0	0.948526	-0.960335	0.010045
4	6	0	-0.339055	-1.402071	-0.072745
5	6	0	2.682464	0.539280	-0.011520
6	6	0	3.294553	-0.646398	-0.039451
7	6	0	2.230823	-2.716090	-1.203956
8	6	0	2.474812	-2.591370	1.325717
9	6	0	2.104769	-4.319881	-3.502493
10	6	0	2.767074	-2.257671	-2.412394
11	6	0	1.660085	-3.997913	-1.132833
12	6	0	1.584902	-4.793662	-2.288521
13	6	0	2.708338	-3.057813	-3.559326
14	1	0	3.122713	-2.702961	-4.479913
15	6	0	3.398631	-3.646048	1.343095
16	6	0	2.000348	-2.982697	3.680638
17	6	0	3.657026	-4.336569	2.536882
18	6	0	2.957778	-4.006152	3.705317
19	6	0	1.766535	-2.267261	2.495012
20	6	0	4.720139	-0.596470	-0.022327
21	6	0	5.203459	0.765016	-0.041626
22	16	0	3.818592	1.894476	-0.055176
23	6	0	2.253039	-1.783895	0.025226
24	6	0	2.013265	-5.183715	-4.774937
25	6	0	3.242972	-4.766867	5.015629
26	1	0	3.042834	-4.127600	5.850450
27	1	0	2.615536	-5.631616	5.070244
28	1	0	4.269630	-5.069164	5.036617
29	6	0	-2.728647	-0.539111	-0.021832
30	6	0	-3.346297	0.642793	0.021694
31	6	0	-2.305410	2.713548	1.210019
32	6	0	-2.540280	2.592883	-1.321207
33	6	0	-2.182290	4.322513	3.505455
34	6	0	-2.846112	2.257343	2.418967
35	6	0	-1.739692	3.996240	1.136665
36	6	0	-1.664092	4.794124	2.290192

37	6	0	-2.786985	3.060765	3.564678
38	1	0	-3.201765	2.709021	4.486082
39	6	0	-1.834846	2.270524	-2.491131
40	6	0	-3.729179	4.337439	-2.532062
41	6	0	-2.071337	2.986065	-3.676362
42	6	0	-3.029974	4.008540	-3.701042
43	6	0	-3.469450	3.645564	-1.338306
44	6	0	-4.764937	0.591820	0.004701
45	6	0	-5.237446	-0.756146	0.013516
46	16	0	-3.858015	-1.892585	-0.098240
47	6	0	-2.314317	1.783527	-0.021334
48	6	0	-2.087562	5.188253	4.776974
49	6	0	-3.315499	4.769971	-5.011848
50	1	0	-4.342223	5.072336	-5.032972
51	1	0	-2.688123	5.634864	-5.066274
52	1	0	-3.115316	4.131003	-5.846945
53	6	0	-1.325114	-0.406617	-0.042717
54	6	0	-1.002303	0.963329	0.007259
55	1	0	-1.520884	2.749434	-4.563182
56	1	0	-1.115005	1.479411	-2.480513
57	1	0	-4.463089	5.116508	-2.550451
58	1	0	-3.988414	3.915256	-0.442537
59	1	0	-1.208767	5.761610	2.243450
60	1	0	-3.302808	1.291201	2.467396
61	1	0	1.128663	-5.761032	-2.244707
62	1	0	1.274088	-4.365609	-0.204627
63	1	0	1.449089	-2.745755	4.567113
64	1	0	3.914213	-3.918562	0.446777
65	1	0	4.389989	-5.116173	2.555554
66	1	0	1.135747	-5.794494	-4.729526
67	1	0	1.960704	-4.548039	-5.634804
68	1	0	2.879171	-5.807575	-4.845208
69	1	0	-1.357687	4.362626	0.206798
70	1	0	0.529117	2.441194	0.188194

71	1	0	-1.209390	5.798136	4.729416
72	1	0	-2.952594	5.813300	4.848168
73	1	0	-2.034167	4.553528	5.637482
74	1	0	5.348330	-1.465292	0.003047
75	1	0	1.046207	-1.476206	2.483209
76	1	0	3.220523	-1.290653	-2.458582
77	1	0	-0.585548	-2.442175	-0.143416
78	1	0	-5.399343	1.453160	-0.006234
79	6	0	6.581858	1.162979	-0.100291
80	6	0	7.126020	2.514728	-0.084863
81	6	0	8.535408	2.546645	-0.111734
82	6	0	9.147279	1.298281	-0.109620
83	6	0	-6.599043	-1.141026	0.114022
84	6	0	-7.073629	-2.477747	0.093035
85	6	0	-8.478061	-2.570561	0.093070
86	6	0	-9.158644	-1.336388	0.118450
87	6	0	12.827733	0.104667	-0.035415
88	6	0	13.295146	-1.358468	-0.032501
89	6	0	12.201140	-2.210907	-0.032483
90	6	0	10.880252	-1.390946	-0.035109
91	6	0	11.294070	0.088282	-0.031278
92	6	0	14.595156	-1.841229	-0.034022
93	6	0	14.763703	-3.191081	-0.032878
94	6	0	13.634562	-4.056402	-0.031216
95	6	0	12.361088	-3.552017	-0.031655
96	1	0	15.434116	-1.177518	-0.036074
97	1	0	11.513204	-4.206474	-0.030982
98	9	0	13.823789	-5.391763	-0.029014
99	9	0	16.009992	-3.711608	-0.032972
100	8	0	9.708554	-1.852617	-0.034319
101	6	0	13.617386	1.216244	-0.041575
102	6	0	15.010870	1.094611	-0.046023
103	6	0	13.038826	2.489676	-0.050440
104	7	0	16.154252	1.012685	-0.048090

105	7	0	12.575340	3.541574	-0.057787
106	6	0	-12.877026	-0.129419	0.037996
107	6	0	-13.343937	1.337066	0.038121
108	6	0	-12.249269	2.188666	0.039584
109	6	0	-10.928368	1.362438	0.042772
110	6	0	-11.336553	-0.125443	0.033737
111	6	0	-10.574395	-1.266259	0.025399
112	6	0	-14.647907	1.825905	0.038295
113	6	0	-14.817160	3.177326	0.036590
114	6	0	-13.688363	4.040346	0.035623
115	6	0	-12.412688	3.533592	0.037986
116	1	0	-15.489372	1.165043	0.039855
117	1	0	-11.567517	4.189798	0.038288
118	9	0	-13.878397	5.377606	0.032341
119	9	0	-16.064176	3.699536	0.035795
120	8	0	-9.756968	1.824188	0.049359
121	6	0	-13.676862	-1.230354	0.045411
122	6	0	-15.068737	-1.092663	0.048542
123	6	0	-13.110287	-2.508634	0.053427
124	7	0	-16.211369	-0.995472	0.050678
125	7	0	-12.645889	-3.557888	0.060004
126	1	0	-11.098218	-2.194864	-0.052717
127	6	0	10.548811	1.220200	-0.030510
128	1	0	11.079896	2.143783	0.033937
129	6	0	-8.343743	-4.978889	0.040607
130	6	0	-6.933039	-4.906270	0.042245
131	6	0	-8.987635	-6.223348	0.020437
132	6	0	-6.838057	-7.343872	0.011211
133	6	0	-6.183600	-6.098511	0.024841
134	1	0	-10.056741	-6.270012	0.014746
135	1	0	-6.267576	-8.249941	0.002771
136	1	0	-5.114367	-6.060967	0.022178
137	6	0	8.606410	4.964750	-0.055963
138	6	0	7.190717	4.983441	-0.020371

139	6	0	7.286939	7.444638	0.058237
140	6	0	6.537576	6.246256	0.029581
141	6	0	9.343926	6.169229	-0.008517
142	1	0	6.788634	8.397768	0.085237
143	1	0	5.473312	6.304418	0.053657
144	1	0	10.417913	6.142920	-0.013844
145	7	0	-6.285418	-3.644167	0.062547
146	7	0	-9.116677	-3.808819	0.059563
147	7	0	6.435167	3.755426	-0.036330
148	7	0	9.272369	3.737942	-0.119990
149	7	0	-8.237814	-7.404091	0.009118
150	7	0	8.685717	7.405148	0.044305
151	8	0	7.936349	0.005405	-0.220220
152	8	0	-7.979214	-0.020335	0.285107

Cartesian coordinates of designed molecule $IO1_b$ at MPW1PW91/6-31G level of theory.

Center Number	Atomic Number	Atomic Type	Coordinates (Angstroms) X	Y	Z
1	6	0	1.295691	-0.479068	-0.004896
2	6	0	0.254298	-1.424499	-0.036130
3	6	0	1.032011	0.913429	0.018589
4	6	0	-0.213629	1.424197	0.105776
5	6	0	2.703872	-0.647319	0.064022
6	6	0	3.321934	0.533131	0.068713
7	6	0	2.411359	2.638793	1.182118
8	6	0	2.624269	2.435346	-1.339830
9	6	0	2.401684	4.312347	3.427835
10	6	0	2.934242	2.182947	2.401963
11	6	0	1.929481	3.946678	1.071008
12	6	0	1.910697	4.780427	2.199025
13	6	0	2.930949	3.020148	3.523169
14	1	0	3.330119	2.671988	4.453055
15	6	0	3.597505	3.446912	-1.385694
16	6	0	2.177486	2.771606	-3.705071

17	6	0	3.884047	4.093738	-2.598266
18	6	0	3.174951	3.755433	-3.758387
19	6	0	1.910912	2.102978	-2.500082
20	6	0	4.698668	0.520362	0.049768
21	6	0	5.197095	-0.775859	0.034548
22	16	0	3.866494	-1.985467	0.149513
23	6	0	2.359897	1.679627	-0.018831
24	6	0	2.359321	5.215263	4.675153
25	6	0	3.491919	4.463222	-5.091903
26	1	0	3.275013	3.803583	-5.906451
27	1	0	2.893552	5.345795	-5.178989
28	1	0	4.527993	4.731090	-5.116865
29	6	0	-2.640321	0.709138	0.129691
30	6	0	-3.308489	-0.440815	0.105421
31	6	0	-2.418875	-2.604652	-1.058907
32	6	0	-2.602251	-2.400871	1.471584
33	6	0	-2.394595	-4.289161	-3.305229
34	6	0	-2.936712	-2.154757	-2.281392
35	6	0	-1.932327	-3.917199	-0.947823
36	6	0	-1.906567	-4.754240	-2.075655
37	6	0	-2.926005	-2.996618	-3.402253
38	1	0	-3.321100	-2.650689	-4.333865
39	6	0	-1.885427	-2.078816	2.626379
40	6	0	-3.865471	-4.068714	2.719763
41	6	0	-2.155344	-2.751783	3.827922
42	6	0	-3.156245	-3.733608	3.879514
43	6	0	-3.577661	-3.415888	1.512170
44	6	0	-4.702488	-0.329719	0.065051
45	6	0	-5.133381	1.015599	0.040099
46	16	0	-3.720779	2.121112	0.163450
47	6	0	-2.357137	-1.644035	0.149816
48	6	0	-2.346754	-5.194330	-4.552038
49	6	0	-3.475568	-4.442265	5.211664
50	1	0	-4.512312	-4.708736	5.235843

51	1	0	-2.878696	-5.325540	5.298273
52	1	0	-3.258609	-3.783381	6.026716
53	6	0	-1.248200	0.491055	0.111629
54	6	0	-1.003159	-0.903295	0.073745
55	1	0	-1.598596	-2.513577	4.708116
56	1	0	-1.132484	-1.320916	2.592832
57	1	0	-4.628080	-4.820670	2.756210
58	1	0	-4.108889	-3.686242	0.621767
59	1	0	-1.511558	-5.746255	-1.997806
60	1	0	-3.336421	-1.165991	-2.360463
61	1	0	1.519485	5.772712	2.122875
62	1	0	1.569481	4.306193	0.129966
63	1	0	1.619950	2.529233	-4.585251
64	1	0	4.129020	3.718713	-0.497822
65	1	0	4.645533	4.845166	-2.637902
66	1	0	1.518662	5.874234	4.610468
67	1	0	2.268890	4.607580	5.551958
68	1	0	3.259627	5.789674	4.730766
69	1	0	-1.571117	-4.276908	-0.007141
70	1	0	0.432174	-2.478140	-0.119368
71	1	0	-1.504746	-5.851915	-4.484729
72	1	0	-3.245620	-5.770339	-4.609291
73	1	0	-2.254752	-4.587653	-5.429580
74	1	0	5.309100	1.397142	0.039640
75	1	0	1.161999	1.339515	-2.467226
76	1	0	3.334930	1.192642	2.476325
77	1	0	-0.392808	2.476779	0.156398
78	1	0	-5.367908	-1.160258	0.058324
79	6	0	13.396043	-2.156263	0.075333
80	6	0	14.618926	-1.245036	0.051473
81	6	0	14.219127	0.085454	0.014382
82	6	0	12.666078	0.192418	0.007383
83	6	0	12.155234	-1.252027	0.024733
84	6	0	15.956838	-1.614055	0.060358

85	6	0	16.876214	-0.616348	0.026128
86	6	0	16.456226	0.741641	-0.010666
87	6	0	15.126373	1.074320	-0.012792
88	1	0	16.252200	-2.644752	0.092266
89	1	0	14.816320	2.096616	-0.036381
90	9	0	17.383101	1.720271	-0.045486
91	9	0	18.192789	-0.916071	0.026004
92	8	0	11.977853	1.244638	-0.011297
93	6	0	13.398834	-3.525957	0.128712
94	6	0	14.605986	-4.237620	0.149848
95	6	0	12.192462	-4.246178	0.163981
96	7	0	15.582853	-4.837775	0.167252
97	7	0	11.213507	-4.847048	0.193672
98	6	0	-13.505941	1.851145	-0.039952
99	6	0	-14.573057	0.756025	-0.095946
100	6	0	-13.976104	-0.490626	-0.204439
101	6	0	-12.406522	-0.323553	-0.225250
102	6	0	-12.126181	1.205557	-0.115192
103	6	0	-11.002041	2.020362	-0.077951
104	6	0	-15.951485	0.919884	-0.050310
105	6	0	-16.725710	-0.191459	-0.114960
106	6	0	-16.124830	-1.471721	-0.228188
107	6	0	-14.751532	-1.613634	-0.274408
108	1	0	-16.387449	1.893733	0.033596
109	1	0	-14.313676	-2.587327	-0.363462
110	9	0	-16.920674	-2.563102	-0.291295
111	9	0	-18.071406	-0.074293	-0.071402
112	8	0	-11.557318	-1.248009	-0.315186
113	6	0	-13.712010	3.179938	0.058852
114	6	0	-15.006119	3.692246	0.128375
115	6	0	-12.608766	4.047562	0.090975
116	7	0	-16.058753	4.129113	0.186437
117	7	0	-11.709260	4.760760	0.117598
118	1	0	-11.227497	3.063825	-0.023170

119	6	0	10.935632	-1.814243	0.001787
120	1	0	10.969784	-2.879884	0.037210
121	6	0	7.085209	3.811396	-0.158183
122	6	0	7.604668	2.489797	-0.122534
123	6	0	9.012915	2.423951	-0.120737
124	6	0	9.615975	3.705630	-0.153866
125	16	0	8.396603	4.976482	0.040613
126	6	0	8.938676	0.078614	-0.090023
127	6	0	7.510234	0.177551	-0.090478
128	6	0	8.638263	-2.359112	-0.112810
129	6	0	7.266693	-2.237469	-0.114591
130	6	0	6.705400	-0.980057	-0.067290
131	6	0	9.490684	-1.258549	-0.068309
132	1	0	9.076695	-3.334542	-0.141678
133	1	0	6.654315	-3.110870	-0.149475
134	7	0	6.881588	1.375947	-0.106929
135	7	0	9.670701	1.229723	-0.103976
136	6	0	-7.292231	-3.364051	-0.051070
137	6	0	-7.726423	-2.023705	-0.075472
138	6	0	-9.122013	-1.875954	-0.081393
139	6	0	-9.809701	-3.093109	-0.051725
140	16	0	-8.679813	-4.442504	-0.247752
141	6	0	-8.918330	0.396562	-0.096640
142	6	0	-7.503309	0.241522	-0.082500
143	6	0	-9.469940	1.684594	-0.101076
144	6	0	-8.585656	2.793838	-0.115526
145	6	0	-7.183540	2.622563	-0.102809
146	6	0	-6.648249	1.338140	-0.056572
147	1	0	-8.990124	3.785340	-0.137776
148	1	0	-6.538285	3.472151	-0.118582
149	7	0	-6.937699	-0.968705	-0.077745
150	7	0	-9.703989	-0.679259	-0.099285
151	1	0	-10.865420	-3.184062	0.076753
152	1	0	-6.274660	-3.682006	0.073026

153	1	0	6.052311	4.073467	-0.290784
154	1	0	10.660240	3.886826	-0.283661

Cartesian coordinates of designed molecule $IO2_b$ at MPW1PW91/6-31G level of theory.

Center Number	Atomic Number	Atomic Type	Coordinates (Angstroms) X	Y	Z
1	6	0	-1.284783	0.750057	0.069304
2	6	0	-0.264654	1.698042	0.014423
3	6	0	-1.027514	-0.634844	0.155175
4	6	0	0.196106	-1.130184	0.311976
5	6	0	-2.668743	0.931360	0.110331
6	6	0	-3.304345	-0.241710	0.129031
7	6	0	-2.392806	-2.351986	1.310979
8	6	0	-2.539259	-2.215252	-1.216745
9	6	0	-2.387068	-3.983314	3.591644
10	6	0	-2.948361	-1.887120	2.508270
11	6	0	-1.876487	-3.655569	1.237285
12	6	0	-1.860962	-4.466500	2.384028
13	6	0	-2.948350	-2.701739	3.646526
14	1	0	-3.375005	-2.343444	4.559605
15	6	0	-3.473195	-3.263469	-1.259859
16	6	0	-2.016697	-2.604958	-3.562932
17	6	0	-3.703917	-3.954523	-2.461013
18	6	0	-2.979132	-3.622478	-3.613802
19	6	0	-1.803359	-1.895150	-2.369591
20	6	0	-4.677337	-0.170667	0.079179
21	6	0	-5.120256	1.160531	0.030657
22	16	0	-3.768574	2.316883	0.148462
23	6	0	-2.333641	-1.410888	0.092133
24	6	0	-2.349334	-4.856278	4.860821
25	6	0	-3.240974	-4.373701	-4.936247
26	1	0	-3.029368	-3.726590	-5.762361
27	1	0	-2.610219	-5.236207	-4.988606
28	1	0	-4.266267	-4.678869	-4.976741

29	6	0	2.558008	-0.441314	0.375238
30	6	0	3.268804	0.680711	0.239061
31	6	0	2.428925	2.907594	-0.944833
32	6	0	2.512203	2.738976	1.575640
33	6	0	2.398132	4.570859	-3.216358
34	6	0	2.991465	2.468374	-2.147952
35	6	0	1.924104	4.222082	-0.852209
36	6	0	1.888304	5.043694	-1.997148
37	6	0	2.971626	3.294806	-3.281605
38	1	0	3.391922	2.947416	-4.201470
39	6	0	1.770980	2.442160	2.733226
40	6	0	3.676102	4.505942	2.812022
41	6	0	1.984302	3.172202	3.920882
42	6	0	2.949231	4.189781	3.966722
43	6	0	3.451552	3.793650	1.618017
44	6	0	4.648655	0.445278	0.116130
45	16	0	3.512697	-1.918189	0.546730
46	6	0	2.316868	1.925889	0.252002
47	6	0	2.332321	5.446301	-4.487116
48	6	0	3.211504	4.956493	5.285848
49	1	0	4.237325	5.262121	5.324818
50	1	0	2.581480	5.820534	5.330663
51	1	0	2.999181	4.317609	6.118407
52	6	0	1.204735	-0.194973	0.304853
53	6	0	0.974604	1.189524	0.180361
54	1	0	1.408705	2.949948	4.794481
55	1	0	1.041169	1.661591	2.713261
56	1	0	4.404277	5.291247	2.840055
57	1	0	4.002219	4.052721	0.737975
58	1	0	1.467927	6.028014	-1.940175
59	1	0	3.430183	1.495654	-2.205300
60	1	0	-1.445528	-5.451954	2.338036
61	1	0	-1.487074	-4.028304	0.312698
62	1	0	-1.445069	-2.367861	-4.436147

63	1	0	-4.016442	-3.531855	-0.378199
64	1	0	-4.435320	-4.735210	-2.497458
65	1	0	-1.492410	-5.496505	4.828794
66	1	0	-2.291108	-4.226239	5.724520
67	1	0	-3.236951	-5.450539	4.912256
68	1	0	1.553119	4.593513	0.080628
69	1	0	-0.450604	2.742159	-0.129416
70	1	0	1.469635	6.078769	-4.443121
71	1	0	3.213196	6.049341	-4.551987
72	1	0	2.267190	4.815880	-5.350553
73	1	0	-5.325388	-1.019289	0.070231
74	1	0	-1.077490	-1.110067	-2.339511
75	1	0	-3.371305	-0.905703	2.553517
76	1	0	0.384813	-2.176988	0.421443
77	1	0	5.379823	1.207652	-0.036757
78	6	0	-7.284678	-3.303974	0.019146
79	6	0	-7.704914	-1.962172	-0.018391
80	6	0	-9.097532	-1.793800	-0.062750
81	6	0	-9.800464	-3.010267	-0.055950
82	16	0	-8.685845	-4.358580	0.216458
83	6	0	-8.836420	0.486184	-0.131374
84	6	0	-7.451580	0.300415	-0.077542
85	6	0	-7.106731	2.675613	-0.168238
86	6	0	-6.602669	1.380340	-0.068398
87	6	0	-9.326548	1.795214	-0.192086
88	1	0	-6.443481	3.516741	-0.193377
89	7	0	-6.903308	-0.918825	-0.026683
90	7	0	-9.652970	-0.577067	-0.120823
91	1	0	-6.269028	-3.635602	-0.064176
92	1	0	-10.854324	-3.106859	-0.199156
93	6	0	7.041310	3.440127	-0.096151
94	6	0	7.524078	2.128668	-0.072547
95	6	0	8.919195	2.010634	-0.048765
96	6	0	9.565787	3.244828	-0.026786

97	16	0	8.395799	4.552608	-0.275244
98	6	0	8.756528	-0.251560	-0.007669
99	6	0	7.360910	-0.097833	-0.002106
100	6	0	9.277422	-1.544608	0.025514
101	6	0	7.037018	-2.451055	0.061242
102	6	0	6.518975	-1.149905	0.074690
103	1	0	6.391462	-3.305511	0.065521
104	7	0	6.770844	1.078321	-0.057824
105	7	0	9.529647	0.826585	-0.032364
106	6	0	4.963661	-0.954366	0.223025
107	1	0	10.612521	3.373852	0.123658
108	1	0	6.007292	3.719062	-0.011929
109	6	0	-13.322041	1.687492	-0.205264
110	6	0	-14.220912	0.445871	-0.176076
111	6	0	-13.448778	-0.707916	-0.129556
112	6	0	-11.932724	-0.328530	-0.126364
113	6	0	-11.872910	1.221319	-0.184626
114	6	0	-15.610171	0.398829	-0.192013
115	6	0	-16.204842	-0.823584	-0.162208
116	6	0	-15.413937	-2.002533	-0.113260
117	6	0	-14.038657	-1.933063	-0.095829
118	1	0	-16.194239	1.295054	-0.226975
119	1	0	-13.453452	-2.827551	-0.056882
120	9	0	-16.028432	-3.207427	-0.084498
121	9	0	-17.554659	-0.914015	-0.180087
122	8	0	-10.961919	-1.129715	-0.084966
123	6	0	-13.710445	2.979663	-0.241898
124	6	0	-15.067889	3.319337	-0.268394
125	6	0	-12.734489	3.984151	-0.251841
126	7	0	-16.176598	3.608084	-0.290297
127	7	0	-11.934253	4.804031	-0.259904
128	6	0	-10.836111	2.119179	-0.216348
129	1	0	-11.096570	3.155190	-0.261990
130	6	0	13.183096	-2.163682	-0.174460

131	6	0	14.461993	-1.356934	-0.284451
132	6	0	14.181655	0.000765	-0.226143
133	6	0	12.609296	0.201420	-0.071406
134	6	0	12.002612	-1.251258	-0.051529
135	6	0	10.790030	-1.934919	0.030244
136	6	0	15.745385	-1.861683	-0.427345
137	6	0	16.769197	-0.983851	-0.516086
138	6	0	16.513224	0.411178	-0.453432
139	6	0	15.220222	0.899887	-0.305837
140	1	0	15.918671	-2.917312	-0.468515
141	1	0	15.052161	1.955670	-0.258713
142	9	0	17.560110	1.265136	-0.542159
143	9	0	18.033196	-1.445287	-0.665753
144	8	0	12.001225	1.302971	0.014130
145	6	0	13.033790	-3.492102	-0.186393
146	6	0	14.128955	-4.341037	-0.323224
147	6	0	11.734193	-4.002456	-0.058035
148	7	0	15.023605	-5.043102	-0.435216
149	7	0	10.663217	-4.398039	0.048355
150	1	0	10.932177	-2.992297	0.105876
151	7	0	-8.453274	2.846804	-0.229381
152	7	0	8.393932	-2.605148	0.046286

Cartesian coordinates of designed molecule $IO3_b$ at MPW1PW91/6-31G level of theory.

Center	Atomic	Atomic	Coordinates (Angstroms)		
**Number**	**Number**	**Type**	**X**	**Y**	**Z**
1	6	0	-1.297759	0.972140	0.069419
2	6	0	-0.281826	1.924283	-0.001996
3	6	0	-1.041842	-0.414140	0.163032
4	6	0	0.181585	-0.900975	0.314415
5	6	0	-2.675107	1.154656	0.104601
6	6	0	-3.313969	-0.017138	0.138679
7	6	0	-2.402779	-2.113749	1.356509
8	6	0	-2.560867	-2.021447	-1.173923

9	6	0	-2.390783	-3.702358	3.670130
10	6	0	-2.958797	-1.628941	2.545879
11	6	0	-1.881641	-3.418357	1.308750
12	6	0	-1.862379	-4.206540	2.472131
13	6	0	-2.955230	-2.421630	3.699965
14	1	0	-3.382041	-2.046911	4.606374
15	6	0	-3.501138	-3.066604	-1.198206
16	6	0	-2.041444	-2.456766	-3.512445
17	6	0	-3.737323	-3.776412	-2.388265
18	6	0	-3.010262	-3.469026	-3.546017
19	6	0	-1.823940	-1.727011	-2.332066
20	6	0	-4.683264	0.062450	0.088440
21	6	0	-5.114646	1.393230	0.022047
22	16	0	-3.760743	2.548497	0.123511
23	6	0	-2.347691	-1.192299	0.118930
24	6	0	-2.351632	-4.551404	4.956177
25	6	0	-3.276201	-4.241830	-4.855036
26	1	0	-3.058289	-3.611352	-5.692272
27	1	0	-2.652249	-5.110134	-4.890921
28	1	0	-4.303845	-4.539543	-4.891616
29	6	0	2.525051	-0.216290	0.360806
30	6	0	3.243884	0.897760	0.229812
31	6	0	2.396884	3.117280	-0.997992
32	6	0	2.502808	2.998874	1.527347
33	6	0	2.358688	4.732169	-3.308519
34	6	0	2.959859	2.655540	-2.192653
35	6	0	1.885998	4.433410	-0.934958
36	6	0	1.840803	5.227241	-2.101507
37	6	0	2.930653	3.454534	-3.346323
38	1	0	3.350701	3.088285	-4.259258
39	6	0	1.763455	2.724031	2.689341
40	6	0	3.676684	4.781764	2.735432
41	6	0	1.978227	3.472763	3.862770
42	6	0	2.949377	4.483087	3.894125

43	6	0	3.449573	4.052538	1.551882
44	6	0	4.623668	0.633421	0.107343
45	16	0	3.459262	-1.707584	0.534445
46	6	0	2.297184	2.156176	0.216123
47	6	0	2.298756	5.582391	-4.596050
48	6	0	3.215678	5.268439	5.203018
49	1	0	4.244586	5.566538	5.239205
50	1	0	2.593147	6.137884	5.235554
51	1	0	2.998365	4.642847	6.044632
52	6	0	1.180453	0.038577	0.300255
53	6	0	0.953459	1.421841	0.165035
54	1	0	1.402486	3.265956	4.738570
55	1	0	1.028010	1.946439	2.679534
56	1	0	4.409811	5.561651	2.754518
57	1	0	3.997489	4.298762	0.665770
58	1	0	1.414568	6.210787	-2.067350
59	1	0	3.399865	1.682660	-2.231551
60	1	0	-1.442477	-5.190864	2.446328
61	1	0	-1.491614	-3.809170	0.391628
62	1	0	-1.468034	-2.238715	-4.389066
63	1	0	-4.045087	-3.317834	-0.312083
64	1	0	-4.474574	-4.552495	-2.412225
65	1	0	-1.492915	-5.189896	4.937262
66	1	0	-2.296044	-3.905264	5.808146
67	1	0	-3.237726	-5.147009	5.017715
68	1	0	1.510443	4.824047	-0.012718
69	1	0	-0.472078	2.966388	-0.149089
70	1	0	1.437151	6.216210	-4.568930
71	1	0	3.181336	6.184397	-4.666919
72	1	0	2.239688	4.935841	-5.447068
73	1	0	-5.339740	-0.778195	0.088720
74	1	0	-1.093828	-0.945654	-2.316289
75	1	0	-3.385633	-0.648523	2.572543
76	1	0	0.383408	-1.945943	0.426650

77	1	0	5.369304	1.375855	-0.020297
78	6	0	-6.801875	-3.106078	0.010261
79	6	0	-7.348870	-1.816628	-0.028459
80	6	0	-8.751550	-1.792816	-0.064538
81	6	0	-9.336507	-3.065501	-0.047224
82	6	0	-8.744718	0.504318	-0.148753
83	6	0	-7.336828	0.470232	-0.103125
84	6	0	-7.218461	2.877936	-0.213982
85	6	0	-6.598162	1.637760	-0.109125
86	6	0	-9.394501	1.749789	-0.212768
87	1	0	-6.644229	3.782355	-0.254460
88	7	0	-6.657329	-0.692583	-0.046674
89	7	0	-9.432496	-0.644618	-0.124837
90	1	0	-5.761966	-3.331601	-0.081492
91	1	0	-10.381052	-3.255165	-0.184308
92	6	0	6.887741	3.350779	-0.148905
93	6	0	7.370909	2.030992	-0.099679
94	6	0	8.764451	1.910063	-0.075520
95	6	0	9.414251	3.125105	-0.076071
96	6	0	8.632185	-0.335366	0.002598
97	6	0	7.230089	-0.198777	0.001614
98	6	0	9.190463	-1.605316	0.059141
99	6	0	6.880078	-2.547313	0.091649
100	6	0	6.380362	-1.248019	0.087111
101	1	0	6.226154	-3.399724	0.099434
102	7	0	6.629254	0.975131	-0.062356
103	7	0	9.382874	0.741018	-0.043150
104	6	0	4.925101	-0.766878	0.213879
105	1	0	10.460113	3.233892	0.073692
106	1	0	5.852198	3.652635	-0.073435
107	6	0	-13.337023	1.222136	-0.199031
108	6	0	-14.104152	-0.101443	-0.157547
109	6	0	-13.220453	-1.174885	-0.109049
110	6	0	-11.746903	-0.657111	-0.116135

111	6	0	-11.854125	0.904642	-0.185424
112	6	0	-15.485238	-0.285911	-0.165665
113	6	0	-15.959976	-1.562092	-0.125861
114	6	0	-15.056080	-2.655403	-0.075324
115	6	0	-13.692934	-2.449213	-0.065727
116	1	0	-16.157341	0.546607	-0.203438
117	1	0	-13.027716	-3.283608	-0.026464
118	9	0	-15.548215	-3.917884	-0.037898
119	9	0	-17.297935	-1.787655	-0.136411
120	8	0	-10.703115	-1.378026	-0.074819
121	6	0	-13.849604	2.470347	-0.241067
122	6	0	-15.232311	2.670028	-0.261700
123	6	0	-12.980953	3.570777	-0.262630
124	7	0	-16.362797	2.861443	-0.279305
125	7	0	-12.283795	4.478682	-0.280599
126	6	0	-10.935378	1.922442	-0.231224
127	1	0	-11.328135	2.931578	-0.283224
128	6	0	13.066629	-2.197978	-0.142586
129	6	0	14.350976	-1.411938	-0.272201
130	6	0	14.087463	-0.046441	-0.236491
131	6	0	12.517697	0.179659	-0.077011
132	6	0	11.910530	-1.265982	-0.023894
133	6	0	10.720187	-1.955110	0.079293
134	6	0	15.627379	-1.937714	-0.414604
135	6	0	16.664274	-1.075680	-0.522019
136	6	0	16.429210	0.321773	-0.483278
137	6	0	15.141748	0.832348	-0.340182
138	1	0	15.786799	-2.996668	-0.440284
139	1	0	14.993394	1.890011	-0.313229
140	9	0	17.492133	1.156858	-0.594561
141	9	0	17.921523	-1.556101	-0.671272
142	8	0	11.907977	1.281895	-0.008253
143	6	0	12.892792	-3.530079	-0.133721
144	6	0	13.980682	-4.388585	-0.262258

145	6	0	11.587784	-4.041165	0.005405
146	7	0	14.872675	-5.096038	-0.367690
147	7	0	10.518612	-4.446185	0.119603
148	1	0	10.909942	-2.997790	0.188492
149	7	0	-8.622960	2.918521	-0.262991
150	7	0	8.285359	-2.718834	0.096199
151	8	0	8.256717	4.438810	-0.348920
152	8	0	-8.093266	-4.294078	0.224762
